# Cells with loss-of-heterozygosity after exposure to ionizing radiation in *Drosophila* are culled by p53-dependent and p53-independent mechanisms

**DOI:** 10.1371/journal.pgen.1009056

**Published:** 2020-10-19

**Authors:** Jeremy Brown, Inle Bush, Justine Bozon, Tin Tin Su

**Affiliations:** Department of Molecular, Cellular and Developmental Biology, 347 UCB, University of Colorado, Boulder, CO, United States of America; Geisel School of Medicine at Dartmouth, UNITED STATES

## Abstract

Loss of Heterozygosity (LOH) typically refers to a phenomenon in which diploid cells that are heterozygous for a mutant allele lose their wild type allele through mutations. LOH is implicated in oncogenesis when it affects the remaining wild type copy of a tumor suppressor. *Drosophila* has been a useful model to identify genes that regulate the incidence of LOH, but most of these studies use adult phenotypic markers such as *multiple wing hair* (*mwh*). Here, we describe a cell-autonomous fluorescence-based system that relies on the QF/QS transcriptional module to detect LOH, which may be used in larval, pupal and adult stages and in conjunction with the GAL4/UAS system. Using the QF/QS system, we were able to detect the induction of cells with LOH by X-rays in a dose-dependent manner in the larval wing discs, and to monitor their fate through subsequent development in pupa and adult stages. We tested the genetic requirement for changes in LOH, using both classical mutants and GAL4/UAS-mediated RNAi. Our results identify two distinct culling phases that eliminate cells with LOH, one in late larval stages and another in the pupa. The two culling phases are genetically separable, showing differential requirement for pro-apoptotic genes of the H99 locus and transcription factor Srp. A direct comparison of *mwh* LOH and QF/QS LOH suggests that cells with different LOH events are distinguished from each other in a p53-dependent manner and are retained to different degrees in the final adult structure. These studies reveal previously unknown mechanisms for the elimination of cells with chromosome aberrations.

## Author summary

Radiation such as that used in radiation therapy of cancer can cause chromosome breakage and loss. We describe here a new method to detect cells with chromosome defects that arise after exposure to X-rays. We used this method to investigate the fate of cells as larval tissues develop into adult organs. Our results reveal ways in which cells with chromosome defects are eliminated, providing a deeper understanding of quality control mechanisms that reduce the propagation of cells with defective genomes.

## Introduction

Loss of Heterozygosity or LOH typically refers to a phenomenon in which diploid cells that are heterozygous for a mutant allele lose their wild type allele through mutations. LOH is implicated in oncogenesis when it affects the remaining wild type copy of a tumor suppressor. *Drosophila* has been a useful model to identify genes that regulate the incidence of LOH. These studies used adult phenotypic markers such as *yellow* (y), *multiple wing hair* (*mwh*), and *javelin* (*jv*). *y* and *jv* affect the adult bristle color and shape, respectively, while *mwh* affects the number of hairs per adult body and wing cell. Heterozygous animals appear wild type but an LOH event in a cell that removes the wild type copy produces a homozygous mutant cell, which then shows cell-autonomous changes. These markers have enabled many decades of fruitful studies that require monitoring genome rearrangements (for example, [1]). These studies have, for example: shown that genes that function in meiotic recombination also play a role in miotic genome maintenance [2]; identified new genes that ensure genome stability such as *mus304* [3]; and demonstrated that a then-newly identified *Drosophila* p53 homolog does indeed have a role in protecting the genome by reducing radiation-induced LOH [4]. Because the phenotypes manifest only in late pupa and adult stages, *mwh*, *y* and *jv* cannot be used to monitor LOH in earlier stages of development. Thus, we have little idea about how cells with LOH behave between the time of LOH induction, for example, by irradiation in the embryo or the larvae, and the time of phenotypic manifestation in the adult. In this regard, it would be useful to have cell-autonomous markers of LOH that are detectable at all stages of development.

One such marker is a fluorescence-based marker for LOH that relies on the ability of GAL80 to repress the transcription factor GAL4. Here, GAL4 drives the expression of GFP but is repressed by one copy of GAL80. An LOH event that eliminates GAL80 de-represses GAL4, resulting in GFP expression. This system was used to detect the appearance of spontaneous LOH in adult *Drosophila* guts [5]. One draw-back of the system, however, is its limited utility. Specifically, it cannot be used in conjunction GAL4/UAS-based inhibition of gene expression, for example, RNAi, to interrogate the role of a gene of interest in LOH induction or elimination.

Apoptosis is one mechanism by which cells with damaged chromosomes are eliminated. In a wide variety of metazoa, agents that damage DNA such as ionizing radiation (IR) also induce apoptosis. IR-induced apoptosis relies on molecules that are conserved in *Drosophila* and human. Apoptosis requires caspase activity, but caspases are normally kept inactive by Inhibitor of Apoptosis Proteins (IAPs). After irradiation in *Drosophila* larvae, counterparts of human SMAC/DIABLO proteins are transcriptionally induced and act to neutralize the IAPs, allowing for caspase activation and apoptosis [6]. The *Drosophila* genome includes four such genes, *hid*, *rpr*, *grim* and *skl*, all of which are located on Chromosome 3R [7, 8]. Studies in *Drosophila* larval imaginal discs, precursors of adult organs, have shown that *hid*, *rpr*, and *skl* are transcriptionally induced by IR (for example, [9]). Larvae that carry mutations in *hid* or *rpr* or a chromosomal deficiency (H99) that removes a copy of *hid*, *rpr*, and *grim* show reduced and delayed IR-induced apoptosis, indicating that *hid* and *rpr* make key contributions to this process.

Studies of IR-induced apoptosis in *Drosophila* larval wing discs have identified both p53-dependent and p53-independent mechanisms. *hid*, *rpr* and *skl* transcriptional induction is maximal at 2-4h after irradiation with an LD50 dose of X-rays (4000R), and this induction requires p53 [9]. As such, p53 homozygous null mutants fail to induce apoptosis at 4-6h after irradiation. At longer times (18-24h) after irradiation, however, p53 mutant discs show robust apoptosis. IR-induced, p53-independent apoptosis requires caspase activity and is reduced/delayed in H99 heterozygotes [9] and *hid* heterozygotes [10, 11]. When p53 null mutants that are also *hid* heterozygotes were irradiated as larvae, the resulting adults show a greater number of aneuploid cells compared to single mutants or wild type [10]. This suggests that p53-independent apoptosis plays a role in elimination of IR-induced aneuploid cells. Aneuploidy in this study was proposed to be segmental aneuploidy (loss of chromosome segment rather than whole chromosomes). Aneuploidy rather than point mutations or mitotic recombination has been identified as the primary mechanism for LOH after irradiation [2]. Because segmental aneuploidy was scored in the adult while irradiation occurred about 10 days earlier in the larvae, it was not possible to determine when in development such cells were eliminated.

In order to understand how cells with IR-induced LOH behave through development, we set out to develop an LOH marker that may be used at all developmental stages. Because we want to use it in conjunction with the GAL4/GAL80 system to modulate gene expression, we chose the QF/QS transcriptional module that originates from the bread mold *Neurospora crassa* but has been shown to work in *Drosophila* [12]. QF is the transcriptional activator analogous to GAL4 and QS is the repressor of QF, analogous to GAL80. The main reason for testing the QF/QS system is the availability of a *Drosophila* line that ubiquitously-expresses QS from a transgene inserted at 100E1, on the right arm of Chromosome 3 [13]. For an LOH marker to be useful, cells that have lost the genetic locus must survive and produce a viable cell or cells that can be scored. It is for this reason that LOH markers in use are close to the telomeres. For example, *mwh* (61F3) is at the tip of 3L and GAL80 (1E) is at the tip of the X chromosome. Segmental aneuploidy that removes *mwh* or GAL80 could remove genes between the respective locus and the telomere, making the cell heterozygous for those genes. Depending on the genes affected, the resulting cell may be at a growth disadvantage. Removal of fewer genes by the event that produced LOH means the greater the chance that cells with the LOH marker survives and can be scored. For this reason, a QS transgene inserted at the tip of 3R is an attractive potential LOH marker.

We report here that using the QF/QS system, we were able to detect the induction of cells with LOH by X-rays in a dose-dependent manner in the larval wing discs, and to monitor their presence through subsequent development in pupa and adult stages. We used a range of X-ray doses up to 4000R that kills about half of the cells in larval imaginal discs [14]. Thus, we are using doses that cause sufficient damage but are still compatible with recovery and regeneration to allow survival [15–17]. We identified two distinct culling steps during development, one in the late larval period and the other in the pupal period. We tested the genetic requirement for changes in LOH cell number, using both classical mutants and GAL4/UAS-mediated RNAi or dominant negative expression, identifying previously unknown mechanisms for the elimination of cells with chromosome aberrations.

## Results

**Fig 1:**
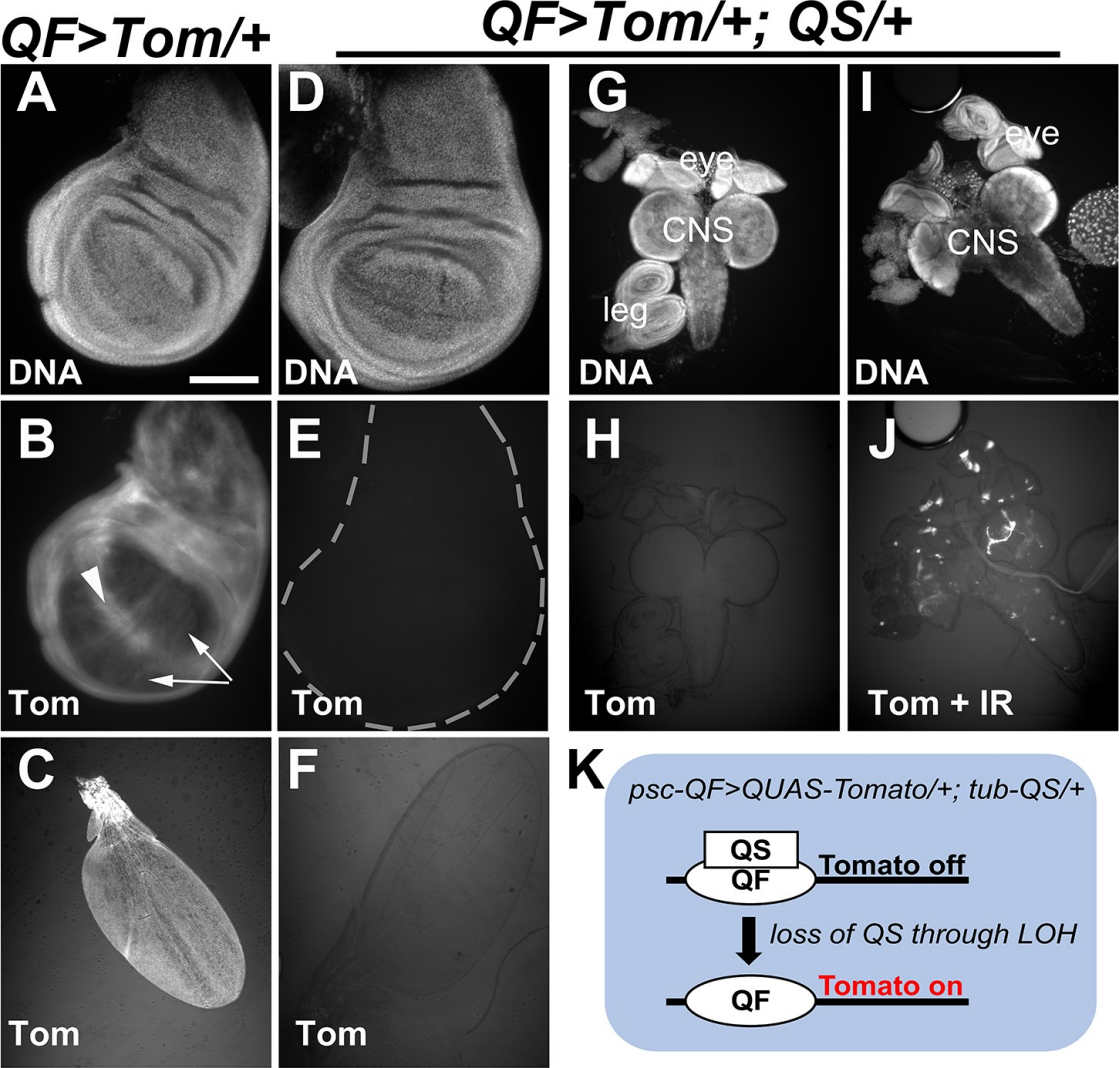
QF>Tom; QS system detects LOH events in irradiated larvae. Larval imaginal discs and Central Nervous System (CNS) were dissected from feeding stage 3^rd^ instar larvae that result from the cross of *w*^*1118*^ with *psc-QF>Tomato or psc-QF>Tomato; tub-QS* (full genotypes provided in S2 Table). The genotypes of the larvae are abbreviated as ‘*QF>Tom/+’* and ‘*QF>Tom/+; QS/+’* respectively. Tissues were fixed, stained for DNA, and imaged. Adult wings were collected from newly eclosed adults within 30 min of unfurling, and imaged. Scale bar = 120 microns in A-B, D-E and 480 microns in C, F, G-J. A-C. *psc*-QF drives the expression of Tomato (QF>Tom) in larval wing discs (B) and a wing from a newly eclosed adult (C). D-H. QF>Tom signal is repressed by one copy of QS in larval wing discs (E), the adult wing (F), and larval discs/CNS (H). The DNA image in D was used to draw the disc outline in E. I-J. QF>Tom signal is de-repressed in some cells of the larval discs and the CNS at 72h after exposure to 4000R of X-rays (J). K. A schematic diagram of the QF>Tom; QS system for detecting LOH.

To develop a fluorescence-based system to monitor LOH, we generated larvae that expressed mtdTomato (Tom) under the control of psc-QF. *psc* (*posterior sex comb*) was chosen for ubiquitous expression in the larval imaginal discs (Fig 1B). Tom signal was observed in the 3^rd^ instar wing disc, with lower intensity in the pouch (Fig 1B, arrows) compared to the rest of the disc and the D/V boundary (arrowhead). Tom signal was also observed in the newly eclosed adult wings (Fig 1C). Tom signal was absent when the larvae carried one copy of the repressor QS expressed from the tubulin promoter (Fig 1E, 1F–1H). In larvae irradiated with 4000R of X-rays, however, spots of Tom were observed throughout larval brains and imaginal discs (Fig 1J). We interpret these data to mean that cells that began as heterozygotes for tub-QS lost QS expression following irradiation, allowing Tom to be expressed. In other words, Tom-positive cells are cells that have lost QS through an LOH event (Fig 1K).

### Dose and larval-age-dependent induction of LOH clones in larval wing discs

**Fig 2:**
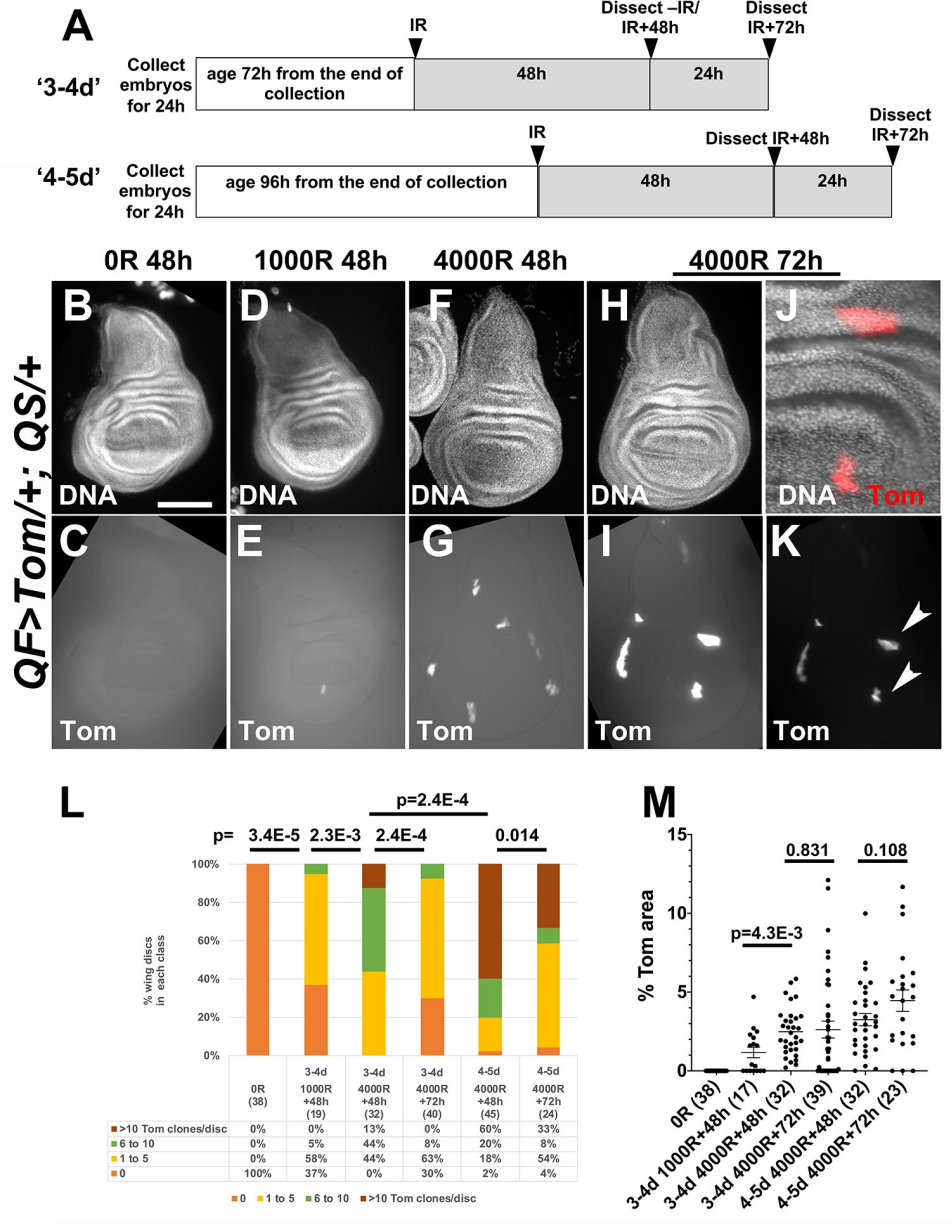
Dose-and larval-age-dependent induction of Tom clones in larval wing discs. A. The experimental protocol. Embryos were collected for 24h and aged for 72h or 96h from the end of the collection to yield larvae that were 3–4 days (d) or 4-5d old at the time of irradiation, respectively. Wing imaginal discs were dissected, fixed, stained for DNA, and imaged. Scale bar = 120 microns in B-I and K and 40 microns in J. B-K. Wing discs from larvae irradiated at 3-4d old with 0R, 1000R or 4000R of X-rays. Time after irradiation is shown above the panels. At 72h after irradiation (I), Tom clones were larger, less numerous and brighter than at 48h after irradiation (G). Panels G and I show identical exposures whereas panel K shows a 4-times lower exposure of the same disc in I. J. A 3X magnified view of two Tom clones indicated with arrowheads in K. L. The number of Tom clones were manually quantified from images such as those in K. The discs were binned according to the number of clones per disc. The number of discs for each bar is shown in parentheses. Kolmogorov–Smirnov test was used to calculate p-values. Additional p-values are shown in S1 Table. M. The total Tom area of each wing disc from the samples in L was quantified and normalized as % of the total disc area. The number of discs for each sample is shown in parentheses. Only discs that were flat (not folded over) were used in this analysis, which is why the disc number is lower in M than in L for some samples. 2-tailed Student t-test was used to calculate p-values. The horizontal bars show the mean±SEM. The numbers of biological replicates were: 2 for *w*^*1118*^+1000R, 4 for 4-5d *w*^*1118*^ IR+48h, and 3 each for all other samples/conditions.

The level of LOH, as detected by the appearance of Tom-positive cells, was X-ray dose- dependent (Fig 2). Embryos were collected for 24h at 25°C and aged for 72h from the end of the collection to produce larvae that were 3–4 days (d) old at the time of irradiation (Fig 2A). We detected no LOH events in unirradiated wing discs, but the number of Tom-positive spots increased at 1000R (Fig 2E) and further increased at 4000R (Fig 2G; quantified in Fig 2L). Tom spots were clearly visible at 48h after irradiation and increased in size and intensity at 72h after irradiation (compare Fig 2G–2I, imaged at the same exposure; K is 4X lower exposure of I). Each spot was composed of many cells (for example, Fig 2J is the magnified view of two clones indicated with arrowheads in K), indicating that cells with QS LOH were capable of proliferation to make clones. Quantification showed that Tom clone number decreased from 48 to 72h after irradiation (Fig 2L, compare the middle two bars). The decrease was statistically significant (p = 2.4E-04, Kolmogorov–Smirnov test, S1 Table). Quantification of Tom area (fractional area of each disc that is Tom^+^) showed dose-dependent induction of LOH (Fig 2M, the first three groups). Tom area, however, was not significantly different between 48 to 72h after irradiation (Fig 2M, middle two groups). In other words, while clone number decreased from 48 to 72h after IR, total clone area remained the same. This is likely because each remaining clone expanded by proliferation between 48 and 72h after IR, which is consistent with the presence of multiple cells per clone (Fig 2J).

The frequency of LOH cells, we found, was dependent on the larval age at the time of irradiation. Larvae that were aged for 96h from the end of embryo collection, that is, 4-5d old at the time of irradiation (Fig 2L, the last two bars), showed more Tom clones than larvae that were 3-4d old at the time of irradiation (see S1 Table for p-values). We do not know the reason for this but such age-dependence was reported for cells with telomere loss induced by FLP/FRT-mediated recombination in larval wing discs; heat-shock induction of FLP produced more clones with telomere loss when applied in older larvae [18]. Larval age-dependent increase in clone number and reduced clone size was also reported for *mwh* clones scored in the adult from irradiation of larvae [2]. In this case, the authors attributed it to the presence of more cells in older larvae. In our studies, although older wing discs showed more Tom LOH clones at 48h after irradiation, their numbers also decreased by 72h after irradiation, similar to the case of younger larvae. And as in the younger larvae, total Tom area did not change significantly (Fig 2M, last two groups), suggesting clonal expansion by cell proliferation also in older larvae.

### LOH clone number and clone size are sensitive to the dosage of pro-apoptotic genes

**Fig 3:**
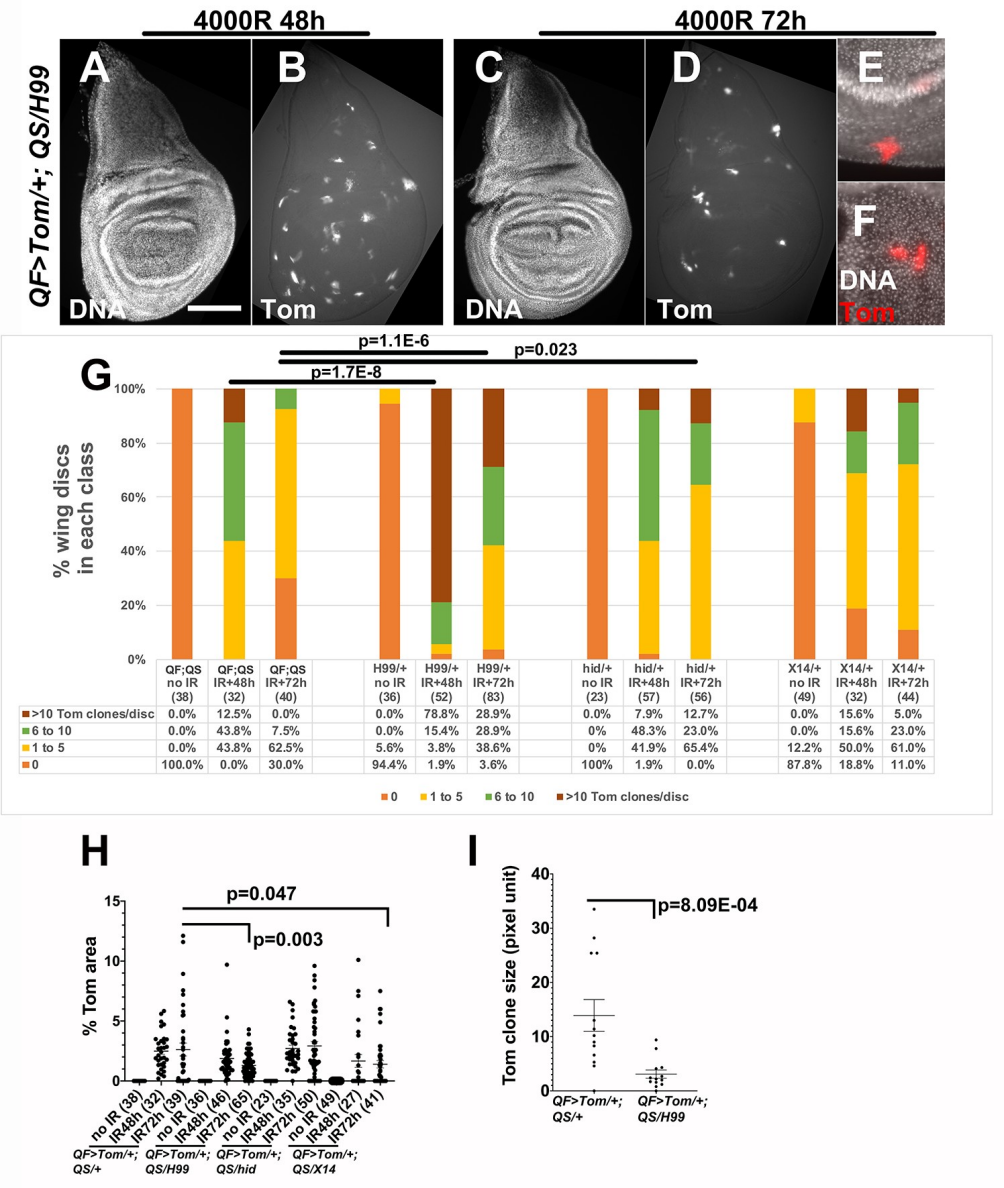
Heterozygosity in pro-apoptotic loci increases the number of Tom clones. Wing imaginal discs were dissected from *psc-QF>Tomato/+; tub-QS/H99* 3^rd^ instar larvae, fixed, stained for DNA, and imaged. The larvae were 3-4d or 4-5d old at the time of irradiation (protocol in Fig 2A). IR = 4000R. Scale bar = 120 microns in A-D and 40 microns in E-F. A-D. Wing discs from *QF>Tom/+; QS/H99* + larvae irradiated with 4000R of X-rays. Time after irradiation is shown above the panels. E-F. Representative clones from a *QF>Tom/+; QS/H99* disc at 72h after IR are shown magnified. G. The number of Tom clones was quantified for wing discs from H99, *hid*^*05014*^, and X14 heterozygotes that also carry one copy each of *QF>Tom* and *QS*. ‘QF;QS’ is *QF>Tom/+; QS/+* controls. The discs were binned according to the number of clones per disc. The number of discs for each bar is shown in parentheses. Kolmogorov–Smirnov test was used to calculate p-values. Additional p-values are shown in S1 Table. H. The total Tom area of each wing disc from the samples in G were quantified and normalized as % of the total disc area. The number of discs for each sample is shown in parentheses. Only discs that were flat (not folded over) were used in this analysis. A 2-tailed Student t-test was used to calculate p-values. I. The area of 12 individual randomly chosen clones per genotype, from 6 control and 7 H99/+ discs, were quantified in ImageJ. A 2-tailed Student t-test was used to calculate the p-value. The horizontal bars in H and I show the mean±SEM. The numbers of biological replicates were: 6 for H99/+ IR+72h and 3 each for all other samples/conditions.

Apoptosis induced by ionizing radiation such as X-rays and γ-rays in larval wing discs initiates at about 4h after irradiation and could be detected for up to 48h after irradiation [9, 19]. To test the hypothesis that apoptosis limits the number of cells with LOH after irradiation, we quantified Tom clones in wing discs from larvae with mutations in pro-apoptotic genes. We used two chromosomal deficiencies, *Df(3L)H99* and *Df(3L)X14*, and *hid*^*05014*^ mutants. H99 removes pro-apoptotic genes *hid*, *rpr* and *grm* along with a number of other genes, while X14 removes *hid* along with a number of other genes [7, 8]. *hid*^*05014*^ is an amorphic allele that results from a p-element insertion between amino acids 105 and 106 [7]. Heterozygotes of each allele have been shown to reduce and delay (but not eliminate) IR-induced apoptosis in multiple studies including our own (for example, [10, 11, 20]). Of the three alleles used, H99 showed the strongest effect; H99/+ discs had more Tom clones than *QF>Tom/+; QS/+* control discs at both 48h and 72h after irradiation (Fig 3B–3D, quantified in Fig 3G). The differences were significant (p = 1.7E-08 at 48h and 1.1E-06 at 72h, Kolmogorov–Smirnov test, S1 Table). The effect of X14 or *hid* mutations was milder and inconsistent; the clone numbers at 48h were similar to controls at 48h and were significantly higher than controls at 72h for *hid* (p = 0.023) but not for X14 (p = 0.968). These data support the hypothesis that apoptosis plays a role in limiting LOH clone number after irradiation. Of the genes deleted by H99, *hid* makes only a partial and inconsistent contribution, suggesting that others (*rpr* and *grm*) also play a role.

We noticed that Tom clones were smaller in H99/+ than in *QF>Tom/+; QS/+* controls (compare Fig 3D to Fig 2I). Quantifications of Tom area (fractional area of each disc that is Tom^+^, Fig 3H) or the size of individual clones (Fig 3I) support this finding. At 72h after irradiation, the effect was strongest for H99 (p = 0.003), weaker but significant for X14 (p = 0.047), and not significant for *hid* (p = 0.637) (Fig 3H). We conclude that while apoptosis appears to limit the number of LOH clones, it appears to promote their increase in size.

### Inhibition of apical caspase Dronc results in reduced Tom clone number and area

We interpret the apparent paradoxical effect of apoptosis on Tom clones in the following way. Limitation of clone number by apoptosis may be a cell autonomous effect, that is, via apoptosis of Tom^+^ LOH cells, with apoptosis resulting from radiation damage or from growth disadvantage and elimination via cell competition ([21] for a recent review). The findings that clone number decreased between 48 and 72h after IR in *QF>Tom/+; QS/+* control discs (Fig 2L) and that H99/+ discs show greater clone number relative to the controls (Fig 3G) support this interpretation. In contrast, the role of apoptosis in clone growth may be a non-cell autonomous effect, which can be explained by the well-documented phenomenon of Apoptosis-induced Proliferation, AiP [22, 23]. AiP is a phenomenon in which cells undergoing apoptosis release mitogenic signals to stimulate proliferation of their neighbors, thereby regenerating the organ/tumor (reviewed in [22, 23]). This phenomenon has been observed in animals ranging from hydra to mammals and is well-documented in *Drosophila* wing discs. Reduced/delayed apoptosis in H99/+ discs could compromise AiP, limiting clone growth. This can explain why Tom clones in H99/+ discs are smaller than in control discs (Fig 3I). We note that interfering with AiP has the potential to not only reduce clone size but also clone number in extreme cases. This is because proliferation of LOH cells is likely necessary to dilute QS and allow LOH cells to become Tom^+^.

**Fig 4:**
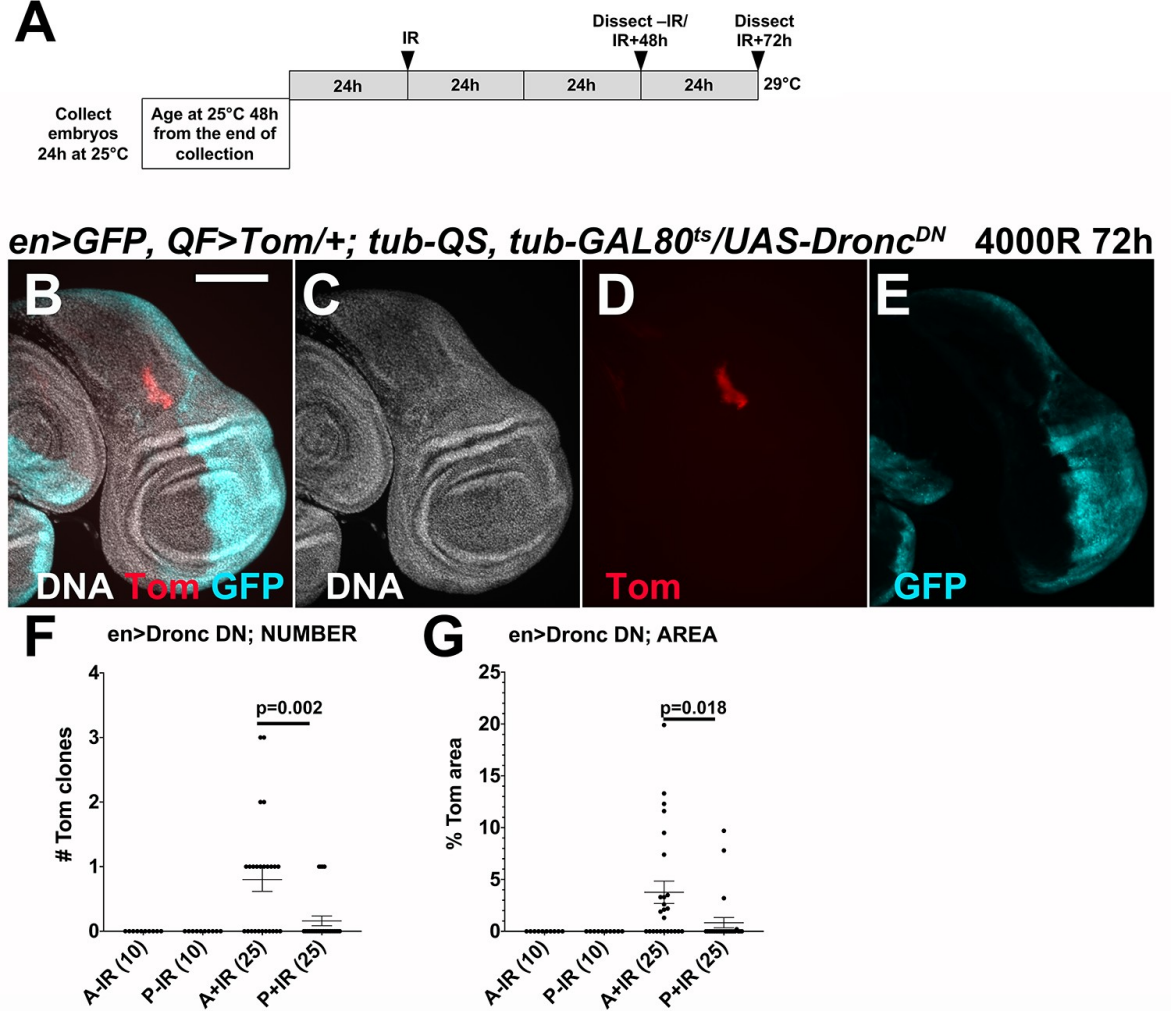
Inhibition of Dronc reduces Tom clones. A. The temperature shift protocol for controlled induction of Dronc^DN^. Embryos were collected for 24h at 25°C, aged for 48h from the end of the collection, shifted to 29°C for 24h, and irradiated. Therefore, larvae were 72-96h old at the time of irradiation. Wing discs were dissected, fixed, stained for DNA, and imaged. Scale bar = 120 microns. All tissues shown are of the genotype *en>GFP*, *psc-QF>Tomato/+; tub-QS*, *tub-GAL80*^*ts*^*/UAS-Dronc*^*DN*^. B-E. A representative wing disc shows a single Tom clone in the anterior. The posterior half that expresses Dronc^DN^ is GFP^+^. F. Quantification of Tom clone number in the anterior (A) and posterior (P) halves. G. Quantification of Tom clone area in the anterior (A) and posterior (P) halves. A 2-tailed Student t-test was used to calculate p-values. The number of discs for each sample in E and F is shown in parentheses. The horizontal bars in E and F show the mean±SEM. The data are from 2 biological replicates per condition.

In *Drosophila* AiP, initiator caspase Dronc is needed to stimulate the production of mitogens, in a role distinct from its role in activating effector caspases to promote apoptosis. To ask if AiP has a role in LOH, we expressed a catalytically inactive mutant of Dronc that was shown before to act as a dominant negative [24]. We expressed Dronc^DN^ in the posterior halves of wing disc using the *en-GAL4* driver (Fig 4). GAL80^ts^ and a temperature shift protocol was used to induce the transgene 24h before irradiation (Fig 4A). Because clone numbers in half discs were lower than in whole discs, we did not bin the discs but report the raw numbers. We find that inhibition of Dronc in the posterior halves of wing discs led to a severe reduction in both the number and the area of LOH clones (Fig 4B–4E, quantified in Fig 4F and 4G). In fact, Tom clones in posterior halves were too few (4 total in 25 discs), preventing meaningful statistical analysis of individual clone size. We conclude that Dronc is needed to generate LOH clones. While the effect of H99/+ (more clones) and Dronc^DN^ (fewer clones) may seem paradoxical, we propose that Dronc^DN^ may be more effective at inhibiting AiP than simply halving apoptotic gene dosage. In the absence of AiP signals in Dronc^DN^ discs, LOH cells may not proliferate sufficiently to dilute the QS protein and appear Tom^+^, leading to reduced clone number. These results support the hypothesis that AiP plays a role in LOH clone growth.

### Changes in LOH clone number during pupa development

**Fig 5:**
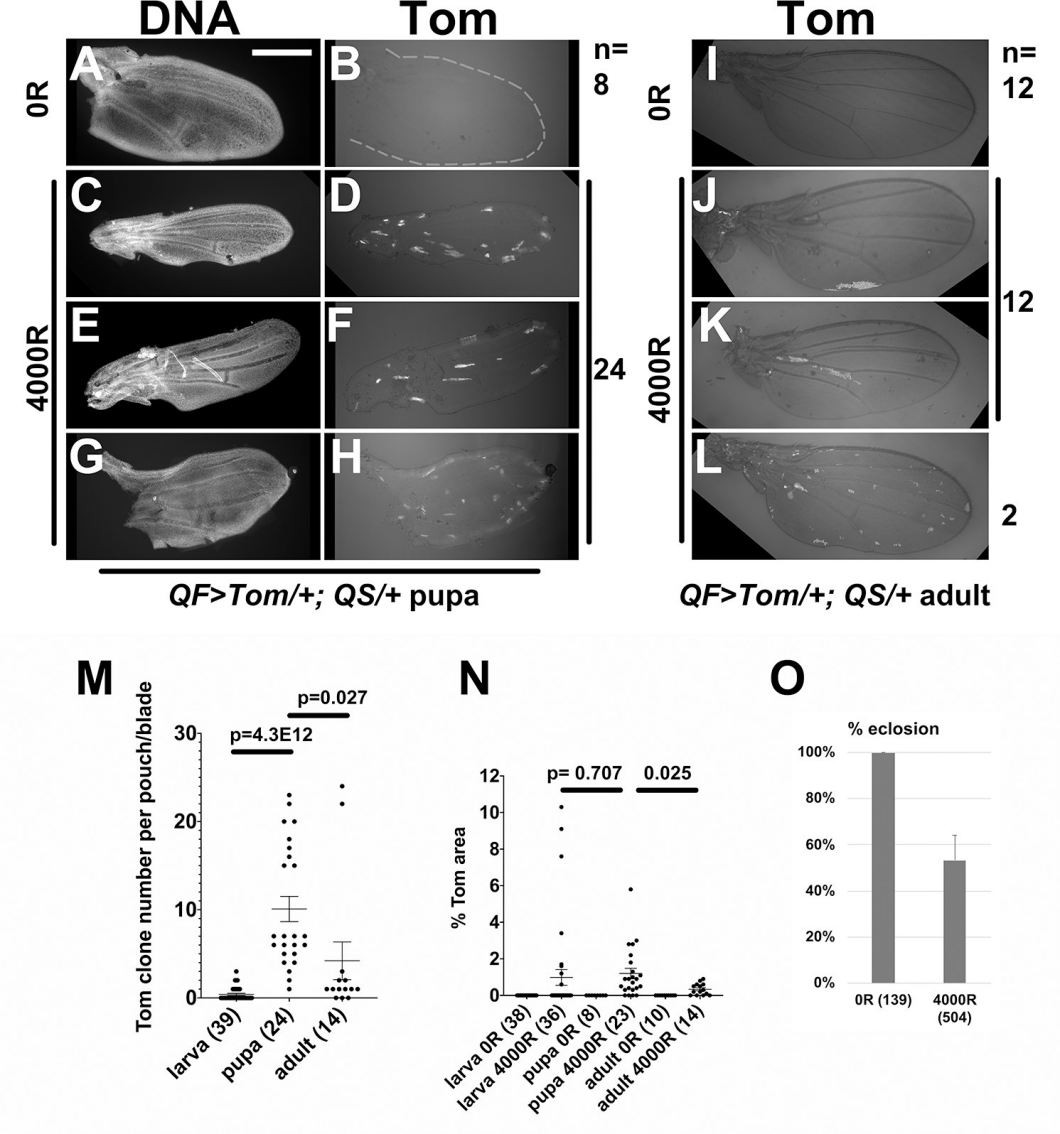
Tom clone number and area decrease during pupariation. 3-4d old *QF>Tom/+; QS/+* larvae were irradiated as in Fig 2A and allowed to develop into pupae. Pupae were collected at the white pupa stage and aged for 24-36h before the wings were dissected, fixed, stained for DNA, and imaged. Adult wings were collected from newly eclosed adults within 30 min of unfurling, and imaged. Scale bar = 240 microns in A-H and 480 microns in I-L. A-H. Representative *QF>Tom/+; QS/+* pupal wings. The number of wings observed is indicated on the side of the panel. The DNA image in A was used to draw the outline in B. I-L. Representative *QF>Tom/+; QS/+* adult wings. The number of wings observed in each category is indicated on the side of the panel. M-N. The number (M) and area (N) of Tom clones at different stages from *QF>Tom/+; QS/+* animals that were irradiated with 4000R as 3-4d old larvae. Clones in the pouch region of larval wing discs from Fig 2 and in the wing blade from images such as those in Fig 5A–5L were quantified. Tom area for each pouch/wing blade is expressed as % of total pouch/wing blade area. The number of wings for each sample in M-N is shown in parentheses. A 2-tailed Student t-test was used to calculate p-values. The horizontal bars show the mean±SEM. O. Percent eclosion of pupae that were irradiated as 3-4d old larvae. The number of animals counted is in parentheses. Error bar = 1STD. The wing data for larvae, pupa and adults are from 3 biological replicates each for +IR samples and 2 biological replicates each for -IR samples. The eclosion data are the averages of 3 biological replicates.

**Fig 6:**
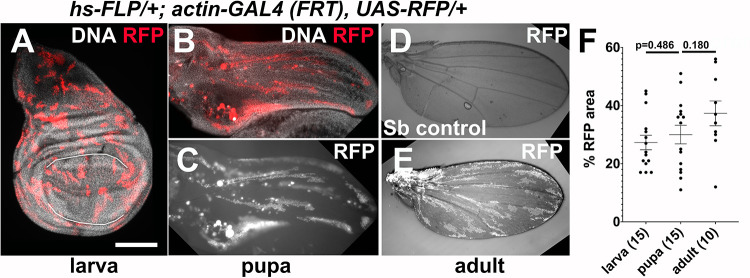
Non-LOH clones are retained from larva to adult. The tissues shown are of the genotype: *Act- FRT-stop-FRT-GAL4>UAS-RFP/+; hs-FLP/+* (A-C, E) and *+/TM3-Sb; hs-FLP/+* (D). 3-4d old larvae were heat-shocked for 30 min at 37°C. The animals were maintained at 25°C except during heat shock. Larval discs were dissected 3d after heat shock, fixed and stained for DNA, and imaged. Pupae were collected at the white pupa stage and aged for 24-36h before the wings were dissected, fixed, stained for DNA, and imaged. Adult wings were collected from newly eclosed adults within 30 min of unfurling and imaged. Scale bar = 120 microns in A, 240 microns in B-C and 480 microns in D-E. A. A representative larval wing disc. The pouch is indicated with white lines. B-C. A representative pupal wing. D. A representative balancer control showing lack of RFP. E. A representative adult wing. F. Total RFP^+^ area of the larval pouch or pupal/adult wing was quantified from images such as those in A-E and expressed as the % of total surface area. The number of discs/wings for each sample is shown in parentheses. A 2-tailed Student t-test was used to calculate p-values. The horizontal bars show the mean±SEM. The data are from 2 biological replicates per condition.

All analyses described thus far are in larval stages. To understand the behavior of LOH clones through development, we examined the wings of *QF>Tom/+; QS/+* pupae and adults that were irradiated as 3-4d old larvae (Fig 5, protocol in Fig 2A). Unirradiated pupa and adult wings lack Tom cells as expected (Fig 5A, 5B and 5I). At 24-36h after pupa formation (APF, measured from white pupa formation), wings from animals that were irradiated as larvae showed Tom clones, with the numbers surpassing those in larval discs (Fig 5D, 5F and 5H show three examples). For quantitative analysis, we compared pupal and adult wing blades to the pouch area of the larval wing disc from which the wing blade develops (see Fig 6A). This showed an increase in both Tom clone number and area from larval to pupal stages (Fig 5M and 5N). The former was statistically significant, but the latter was not. Morphological changes as the wing develops from a disc into a blade likely rearrange the cells of Tom clones to result in an apparent increase in clone number. In contrast, both clone number and Tom area were reduced in the wings of newly eclosed adults compared to the pupa (Fig 5J and 5K, quantified in 5M and 5N). Only 2 of 14 wings examined in two biological replicates retained clone numbers similar to what was seen in the pupa (Fig 5L). The reduction in Tom clone number and area from pupa to adult was statistically significant (Fig 5M and 5N).

The reduction in clone number between 36h APF and eclosion could be because pupae with high clone number failed to eclose and died as pupae. We do not favor this possibility because about half of the pupa eclosed into adults under these conditions (Fig 5O), yet most (12/14) of the eclosed adults have small clone numbers. These data help us rule out the possibility that adults show few clones because pupae with many LOH clones died. Instead, the data support the possibility that some Tom clones were eliminated between 30h APF and eclosion. In contrast, RFP-marked clones that were induced in the larvae by heat-shock-FLP-mediated recombination persist through pupal stages into adulthood without significant reduction (Fig 6). Thus, elimination appears specific to LOH cells.

### Reduction of LOH clones in pupa occurs even with reduced apoptotic gene dosage

**Fig 7:**
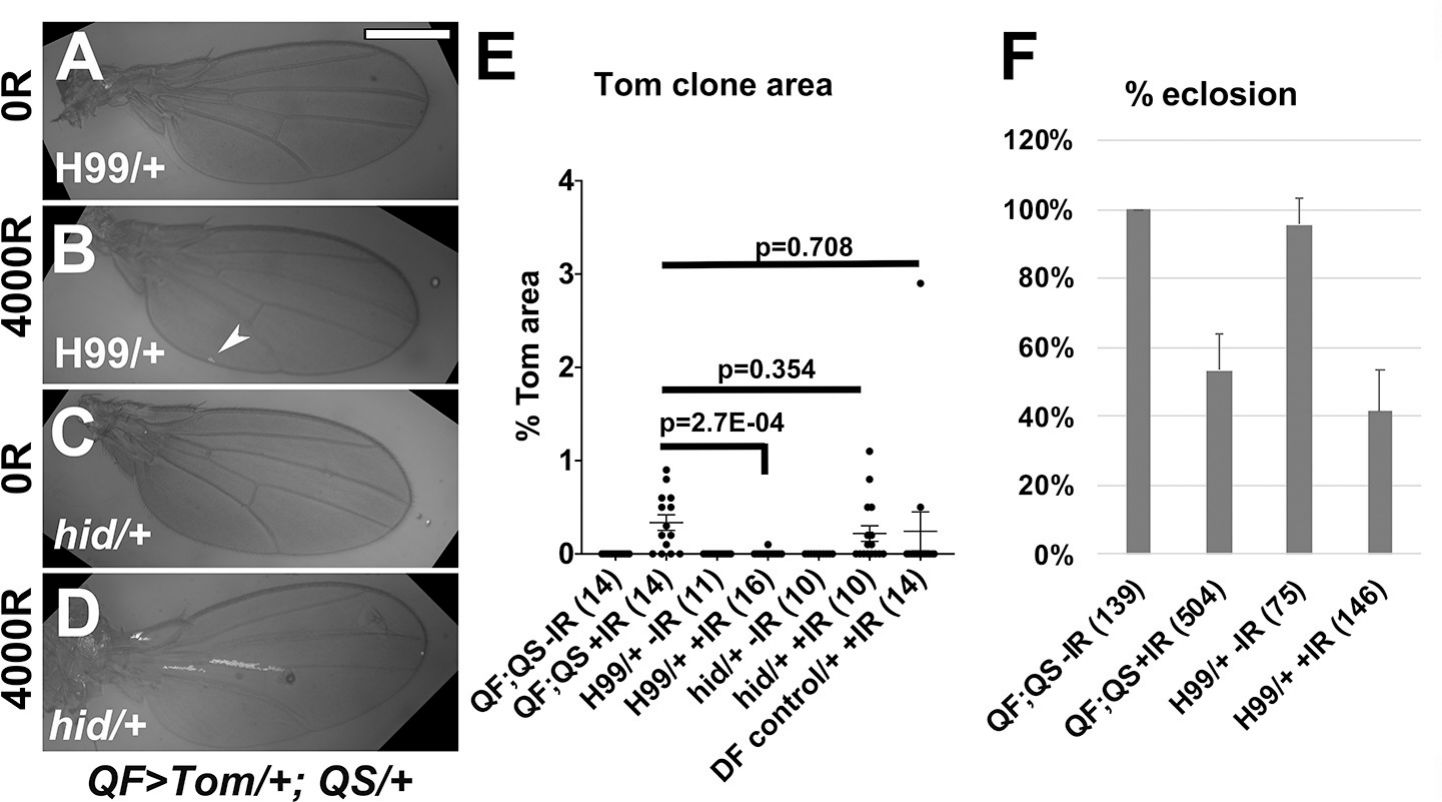
Mutants in apoptotic genes can eliminate LOH clones in the pupa. 3-4d old larvae were irradiated with 0 (-IR) or 4000 (+IR) R of X-rays as in Fig 2A and allowed to develop into adulthood. Adult wings were collected from newly eclosed adults within 30 min of unfurling and imaged. Scale bar = 480 microns. All larvae carried a copy each of *QF>Tom* and *QS* (abbreviated as ‘QF;QS’), in the background of the genotypes shown. A-D. Representative adult wings with the genotypes indicated. The largest clone observed in a H99/+ wing is indicated with an arrow head in B. E. Tom area from each wing blade was quantified and expressed as % of total wing blade area. The number of wings for each sample is shown in parentheses. A 2-tailed Student t-test was used to calculate p-values. The horizontal bars show the mean±SEM. The numbers of biological replicates were: 2 each for *hid/+*, DF control, *QF>Tom/+;QS/+* -IR, and H99/+ +IR; 3 each for all others. F. Fraction of pupae that eclosed into adults from animals that were irradiated as 3-4d old larvae. *QF>Tom/+;QS/+* data from Fig 5O is included for comparison. For each bar, the average from three biological replicates is shown, along with the number of animals counted in parentheses. Error bar = 1STD.

Unlike in larval discs (Fig 3), the reduction of Tom clones in the pupa appears insensitive to the dosage of the apoptosis locus that is removed by H99 deficiency (Fig 7A and 7B, quantified in Fig 7E). In fact, H99/+ wings show near absence of Tom; the arrowhead in Fig 7B points to the largest clone we found in this genotype. As in the case of *QF>Tom/+; QS/+* control pupae, the eclosion rates are high enough to rule out the possibility that death of pupae with high incidence of LOH was the reason for the absence of Tom clones in adult wings; about 40% of H99/+ pupae eclosed (Fig 7F), yet nearly all are devoid of Tom clones in the adult wings. In other words, while H99/+ larvae enter pupariation with LOH clones in the pouch (Fig 3D), they exit pupariation with a near total absence of LOH (Fig 7B). We conclude that Tom clones in the pupae are still eliminated when the pro-apoptotic gene dosage has been halved, a condition that is known to reduce or delay IR-induced apoptosis [9, 20]. *hid*^*05014*^*/+* wings show a similar level of Tom clones as *QF>Tom/+; QS/+* (Fig 7C and 7D, quantified in Fig 7E). This parallels the finding that the effect of *hid*^*05014*^*/+* was weaker than the effect of H99/+ in larval discs (Fig 3G and 3H). The effect of H99/+ is unlikely to be due to non-specific effects of a chromosomal deficiency; an unrelated deficiency (Df(3L)Exel6130, S1 Table) that removes a similar amount of chromosome III as H99 was not significantly different from *QF>Tom/+; QS/+* (‘Df control’ in Fig 7E, p = 0.708).

### Reduction of LOH clones in pupa is sensitive to Srp gene dosage

**Fig 8:**
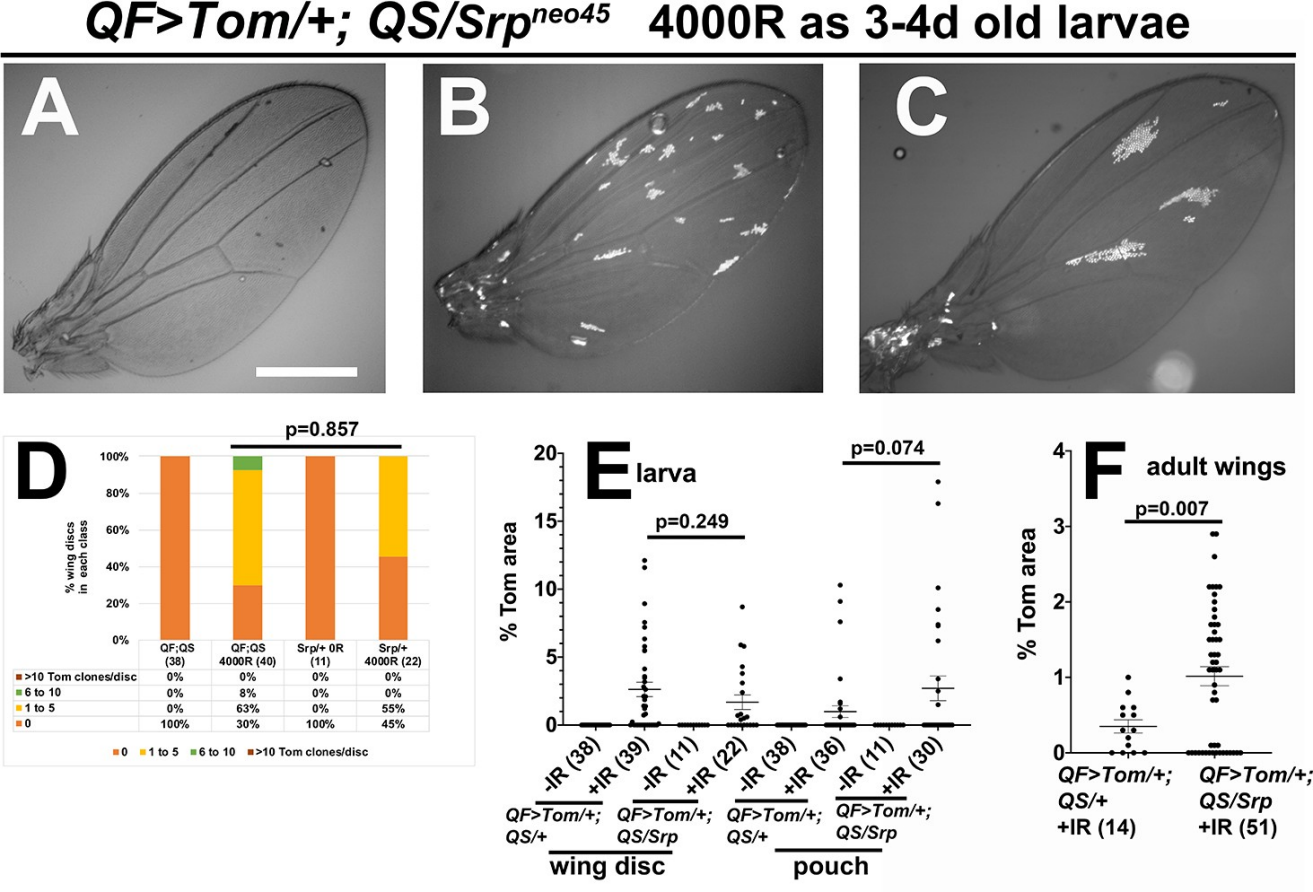
The effect of Srp gene dosage on LOH. 3-4d old larvae of the genotype *psc-QF>Tomato/+; tub-QS*, *tub-GAL80*^*ts*^*/Srp*^*neo45*^ were irradiated with 0 (-IR) or 4000 (+IR) R of X-rays as in Fig 2A. To analyze wing discs, larvae were dissected 48h (-IR) or 72h (+IR) after irradiation. Wing discs were fixed, stained for DNA, and imaged. Adult wings were collected from newly eclosed adults within 30 min of unfurling, and imaged. Scale bar = 480 microns. A-C. Representative adult wings from irradiated larvae show the range of LOH levels observed. D. Wing discs were binned according to the number of Tom clones as in Fig 2L. E-F. The total Tom area of each wing disc (E) or wing (F) was quantified and normalized as % of the total area. *QF>Tom/+;QS/+* data were reproduced here for comparison. A 2-tailed Student t-test was used to calculate p-values. The horizontal bars show the mean±SEM. The data are from 3 biological replicates each for *QF>Tom/+;QS/+* and 2 each for *Srp/+*.

For AiP, Dronc acts through JNK to produce mitogenic signals Wg and Dpp. Dronc activity results also in increased extracellular Reactive Oxygen Species [22, 23]. ROS has multiple downstream effects that promote regenerative proliferation, including the recruitment of hemocytes that secrete JNK ligand Eiger to sustain mitogen production [22, 23] and activation of JAK/STAT signaling via the p38 MAPK pathway [25]. Transcription factor Srp is important for hemocyte development and function. *Srp*^*neo45*^ allele still allows hemocyte to form but prevents their function in a dominant manner [26]. We used *Srp*^*neo45*^ heterozygotes to investigate whether the Srp-ROS-hemocyte axis is important for the formation or maintenance of LOH clones (Fig 8). We saw no significant difference between *QF>Tom/+; QS/+* and *QF>Tom/+; QS/Srp*^*neo45*^ in terms of clone number (Fig 8D), clone area in the whole wing disc (Fig 8E) or clone area in the pouch (Fig 8E). Although control and *Srp*^*neo45*^*/+* larvae enter pupariation with similar Tom clone number and area, they exit pupariation with a difference. In newly eclosed adults, *Srp*^*neo45*^*/+* showed three-fold more LOH area than *QF>Tom/+; QS/+* controls and this difference was significant (Fig 8F). We conclude that Srp-dependent signal amplification in AiP is not important for LOH clone expansion in the larvae but that Srp is important for the culling of LOH cells in the pupa.

### Depletion of p53 by RNAi increases IR-induced LOH as measured by *mwh*

**Fig 9:**
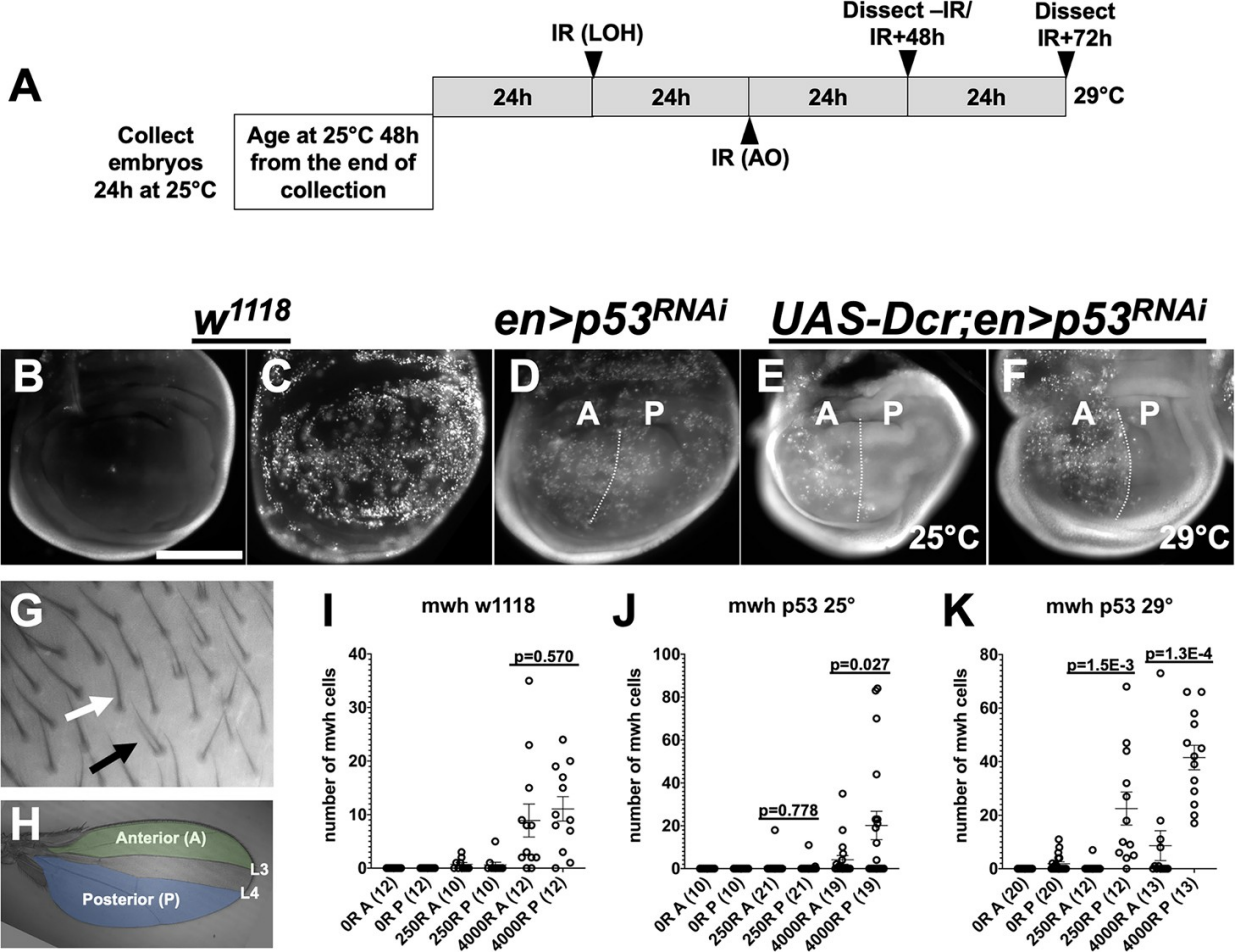
p53 RNAi reduces apoptosis and increases LOH as measured by *mwh*. Wing discs were dissected from feeding 3^rd^ instar larvae at 4h after irradiation, stained with Acridine Orange (AO), and imaged. Adult wings were imaged without fixing or staining to visualize wing hairs. Scale bar = 120 micros in B-F, 60 microns in G and 872 microns in H. A. The temperature shift scheme used in the ‘29°C’ protocol. Embryos were collected for 24h at 25°C, aged for 48h from the end of the collection, shifted to 29°C for 24h, and irradiated. Therefore, in both 25°C and 29°C experiments, larvae were 72-96h old at the time of irradiation. 29°C larvae were kept at 29°C after irradiation for the remainder of the experiment. B-C. AO stain shows robust apoptosis in *w*^*1118*^ wing discs at 4h after irradiation with 4000R of X-rays. D. Expression of p53^RNAi^ with en-GAL4 driver has a mild effect on apoptosis in the posterior (P) half compared to the anterior (A) half. E-F. The addition of UAS-Dcr to en-GAL4>p53^RNAi^ reduces apoptosis in the posterior halves to background un-irradiated levels. G. An example of a wild type (white arrow) and a *mwh* cell (black arrow). H. The anterior and posterior areas of the adult wing examined for *mwh* are indicated. The A/P boundary lies between veins L3 and L4, but without an independent marker, we cannot tell exactly where the boundary is. Therefore, the area between L3 and L4 was not considered. I-K. The number of *mwh* cells was quantified under different conditions. The number of wings examined for each sample is shown in parentheses. p-values were calculated by a 2-tailed Student t-test. Horizontal lines for each group indicate the mean±SEM. The data are from 2 biological replicates for p53 RNAi -IR samples and p53 RNAi 25° 4000R sample. All others are from 3 biological replicates each.

A p53 loss-of-function classical mutant has been shown before to increase the number of *mwh* cells in adult wings that developed from irradiated larvae [4]. To use p53^RNAi^ for LOH studies, we first tested whether it has an effect on LOH as measured by *mwh*. We used en-GAL4 to deplete p53 in the posterior half of the wing disc and monitored IR-induced apoptosis, a process that is affected by p53 status (for example, [9, 10, 20]). One copy each of the transgenes, en-GAL4 and UAS-p53^RNAi^ reduced but did not eliminate IR-induced apoptosis as assayed by staining with the vital dye Acridine Orange (AO) at 4h after irradiation with 4000R of X-rays (Fig 9C and 9D). The addition of UAS-Dcr improved the effect of p53^RNAi^ as seen by reduced apoptosis in the posterior half of wing discs where en-GAL4 was active (Fig 9E). Maintaining the larvae at 25°C or shifting them to 29°C before irradiation as in Fig 9A produced similar results on apoptosis (Fig 9E and 9F). Because UAS-Dcr improved the efficiency of p53^RNAi^, it was included in all subsequent experiments with p53. In this experimental context, we monitored the appearance of *mwh* cells in *mwh/+* animals through LOH (Fig 9G), in anterior and posterior regions of the adult wing (Fig 9H).

Exposure to IR increased *mwh* cells in a dose dependent manner (Fig 9I). In the *w*^*1118*^ wild type background, IR-induced-*mwh* cell number was similar in anterior and posterior regions of the wing (Fig 9I). With p53^RNAi^, we saw a greater number of *mwh* cells in the posterior compartment relative to the anterior compartment, at both 25°C (Fig 9J) and 29°C (Fig 9K). The difference was greater and more significant (lower p-value) at 29°C than at 25°C. The temperature effect was most obvious at the lower IR dose of 250R, where A/P difference was not seen at 25°C (Fig 9J) but was clearly detectable at 29°C (Fig 9K). The temperature effect can be explained by the fact that wild type GAL4 protein is more active at higher temperatures [27]. The temperature effect, although not discernible by AO stain in Fig 9E and 9F, was discernible by the *mwh* LOH phenotype. We conclude that p53^RNAi^ can be used to increase LOH incidence, especially at 29°C. Therefore, we used the 29°C protocol (Fig 9A) in subsequent experiments.

### The effect of p53 RNAi on LOH as detected by QF/QS is less severe

**Fig 10:**
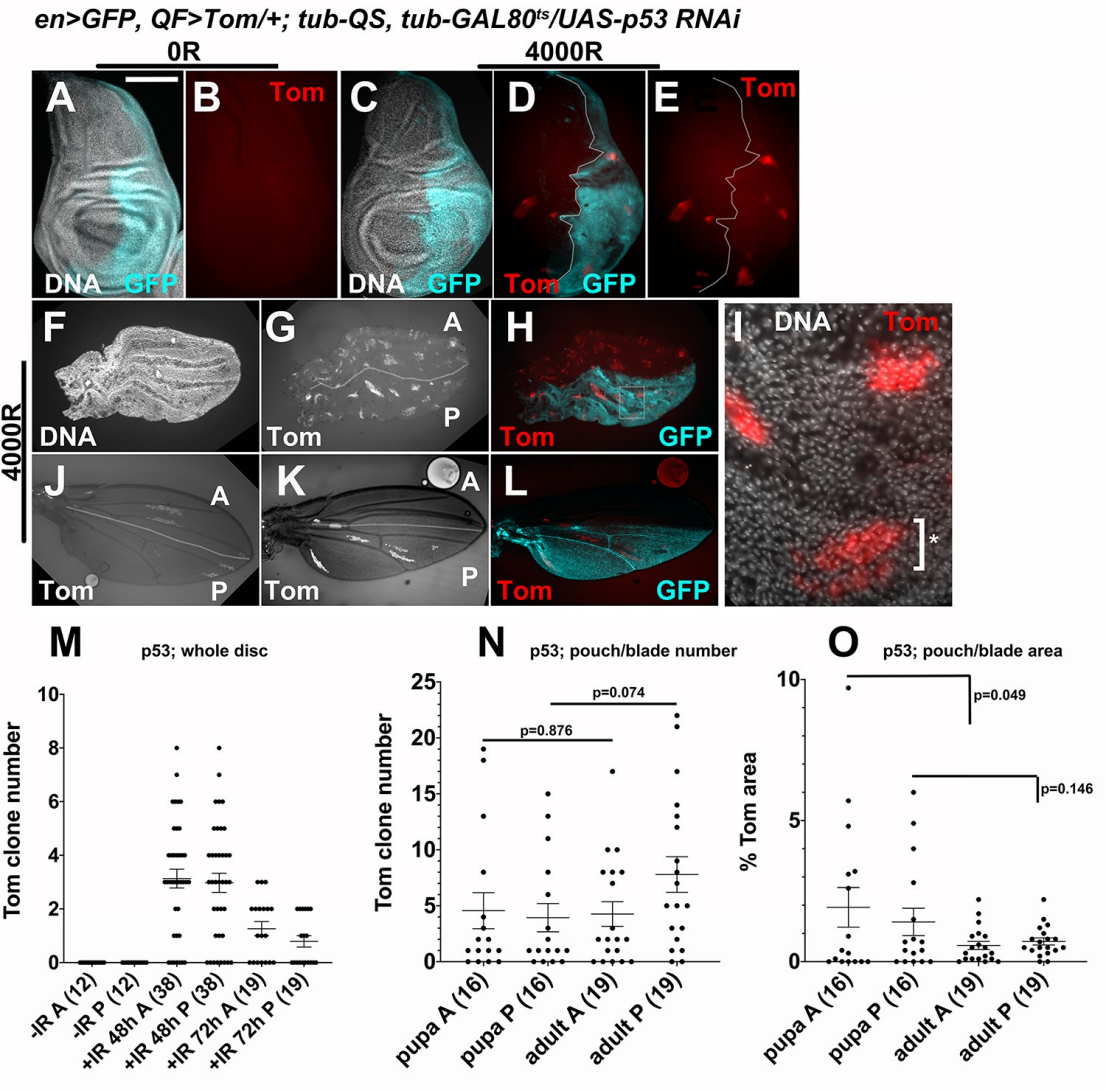
The effect of p53 RNAi on LOH as detected by QF/QS. All tissues shown are of the genotype *UAS-Dcr/+; en>GFP*, *p53RNAi/psc-QF>Tomato; tub-QS/+*. The experimental protocol is as shown in Fig 9A. After the temperature shift, larvae were maintained at 29°C for the remainder of the experiment, that is, until eclosion when adult wings were being studied. Larval wing discs or pupal wings were dissected, fixed, stained for DNA, and imaged. Adult wings were collected from newly eclosed adults within 30 min of unfurling and imaged. Scale bar = 120 microns in A-E, 240 microns in F-H, 480 microns in J-L, and 15 microns in I. A-E. IR induces Tom clones and the clone number is similar in anterior (GFP^-^) and posterior (GFP^+^) halves of larval wing discs. F-H. Tom clone number is similar in anterior (GFP-) and posterior (GFP+) halves of pupal wings. Different channels of the same wing are shown. I. The boxed area in H is shown magnified 4X, to illustrate a ‘broken’ clone (*). J-L. Two representative adult wings, shown to illustrate Tom clones in anterior (GFP^-^) and posterior (GFP^+^) halves. K and L are of the same wing. GFP images were used to mark the A/P boundary (white lines) in J and K. M. Quantification of Tom clone number in larval wing discs at different times after IR. See the text for statistical analysis. N-O. Quantification in pupal and adult wings for clone number (N) and clone area (O). The number of wings examined for each sample is shown in parentheses. p-values were calculated by a 2-tailed Student t-test. Horizontal lines indicate the mean±SEM. The data are from 2 biological replicates each for -IR and IR+48h samples, and 3 each for all others.

Using the QF/QS system with p53^RNAi^, we investigated the effect of reducing p53 expression in the larval discs (Fig 10). We counted Tom clones in the control anterior half and the p53^RNAi^ posterior half, which was marked by UAS-GFP (Fig 10D and 10E, quantified in Fig 10M). Because clone numbers in half discs were lower than in whole discs, we did not bin the discs but report the raw numbers. We saw a statistically significant decrease in clone number between 48h to 72h after IR in both the anterior and the posterior halves (Fig 10M, p = 3.6E-5 for A and 0.004 for P). This is similar to what we saw in *QF>Tom/+; QS/+* control discs in Fig 2. At each time point, however, there was no significant difference between the anterior and the posterior halves (p = 0.752 for 48h and 0.132 for 72h). We conclude that depletion of p53 by RNAi, which was potent enough to eliminate IR-induced apoptosis at 4h after IR (Fig 9E and 9F), did not compromise the reduction in Tom clone number between 48h and 72h after IR. In other words, the reduction in Tom clones in larval stages is p53-independent. This is different from the requirement for pro-apoptotic genes in the H99 locus in the same process (Fig 3). We suggest a reason for this difference in the DISCUSSION.

We next compared Tom clones in p53^RNAi^ pupal and adult wings that result from irradiated larvae, in order to address the role of p53 in the culling of Tom clones in the pupa. We found counting clones in p53^RNAi^ pupa challenging because many appear ‘broken’ or disrupted (for example, * in Fig 10I). We counted the clones nonetheless and saw no significant change from pupa to adult (Fig 10N). In contrast, we could quantify the total Tom^+^ clone area more confidently and found a decrease from pupa to adult in the control anterior halves (Fig 10O), similar to *QF>Tom/+; QS/+* controls (Fig 5N); the difference was significant (p<0.05). In the posterior RNAi half, the decrease was attenuated and no longer significant (p = 0.146). While this could be interpreted as a role for p53 in decreasing clone area in the pupa, we note however, that the effect of p53^RNAi^ on Tom clone area is much weaker than the effect of p53^RNAi^ on *mwh*. Tom areas in A and P halves of adult p53^RNAi^ wings (the last two datasets in Fig 10O) were not significantly different although the same comparison for *mwh* cell number showed a significant difference (the last two datasets in Fig 9K).

### LOH incidence as measured by QF/QS versus *mwh*

**Fig 11:**
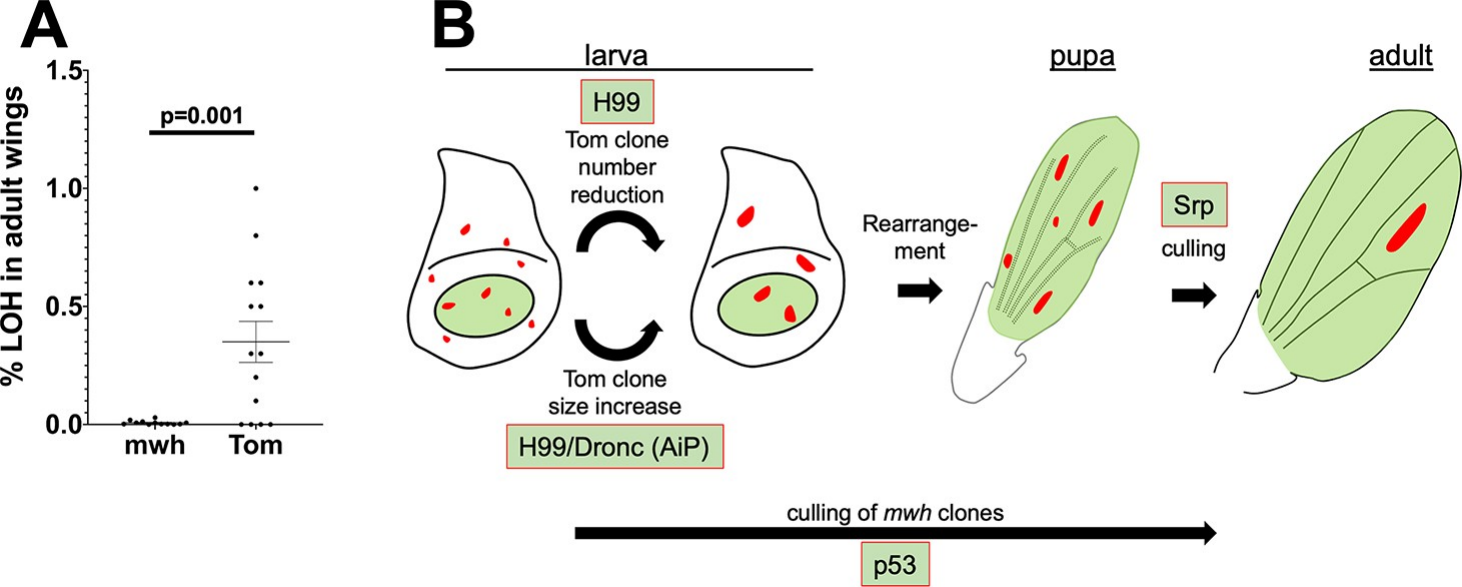
Comparison of LOH by two different detection methods. **A.** Comparison of *mwh* and Tom LOH cell numbers in whole adult wings. Horizontal lines for each group indicate the mean±SEM. The data are from 3 biological replicates each. **B.** Culling of cells with IR-induced LOH during wing development. In larval wing discs, the number of IR-induced QS LOH clones decreases with time after irradiation while the size of remaining clones increases. Full dosage of the H99 locus is needed for clone number decrease while clone size increase, we propose, relies on AiP mediated by Dronc and the H99 locus. Between larval and pupal stages, clone number increases without change in clone area. We attribute this to profound cell arrangements that accompany wing morphogenesis. Both clone number and clone area decrease in subsequent stages of pupariation, and this culling process is sensitive to Srp gene dosage. *mwh* clones are culled though a p53-dependent mechanism between induction in larvae and eclosion, but we do not know exactly when this mechanism acts.

We noticed that in the adult wings, X-rays induced fewer *mwh* cells than Tom cells under the same experimental conditions. To quantify this difference, we converted *mwh* cell number to % of total using the published cell count for the wing blade area of interest (12,200 per wing surface from [1]; see Methods for more details). The results for 4000R are shown in Fig 11A. The difference in *mwh* and Tom incidence was significant (p = 0.001, 2-tailed t-test) and radiation-dependent; *mwh* cells were seen rarely in unirradiated wings and we have not seen any Tom^+^ cells in unirradiated discs/wings. These data indicate that more cells with IR-induced QF/QS LOH survive to contribute to the adult structure than cells with IR-induced *mwh* LOH. Furthermore, the data suggest a contribution by p53 in this difference as noted in a preceding paragraph. In p53^RNAi^ discs irradiated with 4000R using the 29°C protocol, the difference in average LOH between control and p53^RNAi^ halves was 4-fold using *mwh* as the marker (Fig 9K, the last data columns) and 2- or 1.2-fold using Tom clone number or area (Fig 10N and 10O, the last two data columns in each graph), respectively, as the marker. We interpret these data to mean that culling cells with *mwh* LOH shows greater dependence on p53 than culling cells with QS LOH. These data are incorporated into a model (Fig 11B) and discussed further below.

## Discussion

We present a new system for identifying cells with Loss of Heterozygosity (LOH), a widely applicable marker of genome instability. This system is based on fluorescence read-out and may be used in conjunction with the GAL4/UAS system. Unlike the widely used adult markers for LOH, the QF/QS system allows us to observe cells with LOH at larval and early pupal stages. Key observations we made are (Fig 11B):

1. 250-4000R of X-rays applied to 3^rd^ instar larvae induce cells with LOH that are capable of proliferating and contributing to the adult structures.

2. A subset of cells with LOH are culled before the onset of pupariation, in a process that is dependent on *hid* and H99 gene dosage. Because the effect of H99 (which removes *hid*, *rpr*, and *grm*) was greater than the effect of X14 (which removes *hid*) or a *hid* mutant, we suggest that pro-apoptotic genes besides *hid* contribute to culling. This culling is insensitive to p53 depletion.

3. The H99 locus is important for the growth and expansion of LOH clones in the larvae.

4. During pupariation, additional culling occurs such that adult wings bear few cells with LOH even at half lethal doses of X-rays.

5. The requirement for p53 in culling cells with LOH depends on the nature of the LOH event (QS vs. *mwh*).

Observation 1 confirms what is already known using adult markers of LOH (for example, [1, 4]). Observations 2–5 are new knowledge that could not have been gained using adult markers for LOH. We discuss each in detail below.

How do we explain observation 2? We and others have shown that IR induces both p53-dependent and p53-independent apoptosis. p53-dependent apoptosis initiates at about 4h after irradiation with 4000R of IR but p53-independent apoptosis initiates at about 18h after irradiation and could be detected at 72h after irradiation [9]. Both p53-dependent and p53-independent apoptosis are dependent on pro-apoptotic genes uncovered by the H99 deficiency [9, 10]. The first culling of LOH cells occurred at late time points after IR (48-72h), was insensitive to p53^RNAi^, and sensitive to pro-apoptotic gene dosage. Therefore, we suggest p53-independent apoptosis is responsible, at least in part, for this phase of culling. p53-independent-apoptosis was proposed as the mechanism for the elimination of IR-induced segmental aneuploidy (loss of chromosome segments) as detected by an adult body bristle phenotype that results from irradiation of larvae [10]. Therefore, what we are proposing is in good agreement with the published literature and adds to it by identifying the late larval period as the time for this mechanism to eliminate cells with LOH. These data help distinguish the fate the cells with IR-induced LOH ([10] and this study) from that of cells with a broken telomere [18]. The latter, produced in larvae by breakage of di-centric chromosomes, are culled by a p53-dependent mechanism.

Observation 3 poses a paradox. Pro-apoptotic genes, we found, are needed to cull LOH cells in the larvae. Based on these data, one might expect H99/+ mutants to show more LOH cells as adults. H99/+ mutants, however, show fewer LOH cells in the adult wing than *QF>Tom/+; QS/+* only controls. Inhibition of apical caspase Dronc produced an even stronger phenotype in which not only LOH clone area but also clone number was reduced. This is reminiscent of paradoxical findings regarding apoptosis and cancer. Induction of apoptosis, whether by radiation or chemotherapy, is thought to be tumor-suppressive, yet experimental data suggest the opposite. Apoptotic caspase 3 is needed for tumors to regrow after radiation therapy and caspase 3 deficiency in tumors show better response to radiation therapy [28]. This paradox can be explained by Apoptosis induced Proliferation. In mice where a similar phenomenon called Phoenix Rising operates, effector caspase 3 is needed to stimulate the production of mitogenic Prostaglandin E(2) [29]. Caspase 3 -/- mice or tumors are unable to stimulate mitogenic signals after radiation damage and therefore show better response to radiation therapy. Such mice are also defective in wound repair and liver regeneration. H99/+ mutants, we find, may be defective for the initial culling of cells with LOH but are also defective for their clonal expansion, which could occur indirectly through AiP. The overall effect is the reduced incidence of cells with genomic instability, much like in caspase 3 mutant mice where the overall effect is tumor-suppressive. Clonal expansion, we propose, can affect not only clone size but also clone number because proliferation of LOH cells may be required to dilute QS and allow LOH cells to express Tom. Thus, with severe inhibition of AiP, clones may be not only smaller but also fewer, which is exactly what we see with Dronc inhibition. The finding that larval culling is not sensitive to Srp gene dosage suggests that downstream effectors of ROS other than macrophages are involved in this process in the larvae.

What is the mechanistic basis for observation 4? Our data suggest that the second culling step is apoptosis-independent but Srp-dependent (Fig 11B). H99 and *hid* heterozygotes that show reduced/delayed IR-induced apoptosis show adult wings that look similar to *QF>Tom/+; QS/+* (in the case of *hid*/+) or have even fewer LOH cells (in the case of H99/+). The latter observation can be explained as least in part by the finding that H99/+ larval discs already show reduced LOH area and smaller clone size compared to *QF>Tom/+; QS/+*, even before initiating pupariation (Fig 3H–3I). The importance of Srp gene dosage in pupa culling is noteworthy because it suggests a role for hemocytes in removing cells with LOH. A recent study in live *Drosophila* pupa saw circulating hemocytes extravasating from pupal wing veins to sites of damage in the wing epithelium where they are proposed to promote wound-healing and regeneration [30]. It would be interesting to ask if hemocytes also target LOH cells in the pupa and whether additional genes known for hemocyte function are needed for pupal culling.

Observation 5 was an unexpected finding that we were able to make by developing the QF/QS assay for LOH. Besides its role in apoptosis, *Drosophila* p53 is known to have multiple non-apoptotic roles including a delay of larval development in response to IR damage [19], stimulation of Apoptosis-induced-Proliferation in a manner that is independent of its apoptosis-inducing function [31], reprogramming cellular metabolism during cell competition [32], and restraining transposon mobility in the germline [33]. A recent study found that in post-mitotic cells of the adult *Drosophila* head, p53 regulates transcription programs for DNA repair, proteolysis and metabolism in response to IR [34]. We do not know what aspect of p53 functions is responsible for culling *mwh* cells nor do we know when in development this culling occurs because we cannot monitor *mwh* except in wings near the end of development. We do know that in the presence of p53, cells with *mwh* LOH are more efficiently culled than cells with QS LOH; more of the latter are retained in the adult wing. We suggest that the difference in the location of *mwh* and QS relative to the telomere can explain the difference in the retention. The basis of this explanation lies in the finding that aneuploidy is likely to be the primary mechanism for LOH after irradiation [2]. In this study, larvae were irradiated with 1000R of X-rays and LOH examined in the adult abdomen. 29% of LOH clones were twin clones (likely products of mitotic recombination) while 71% were single clones. The *minute* bristle phenotype of cells in single clones suggested that such LOH clones were due to aneuploidy making the cell heterozygous for ribosomal protein genes. These studies did not distinguish various types of aneuploidy (chromosome loss, segmental aneuploidy, terminal deletions). There are 173 sequence-mapped genes between *mwh* and the telomere (3L:61A-61F3) whereas only 5 genes reside between the QS insertion at 100E1 (see Materials and Methods for the insertion sequence) and the telomere (*CG2053*, *krz*, *mod*, *Map205*, and the pseudogene *CR46418*). X-rays induce DNA double-strand breaks (DSB). For a cell to lose *mwh* through a DSB means also losing a copy each of 173 genes whereas cells with QS LOH would be heterozygous for just five genes. Further, *mwh* encodes an actin-binding protein that regulates the actin cytoskeleton [35]. Therefore, becoming a homozygous mutant for *mwh*, whether through aneuploidy, mutations within the gene or mitotic cross-over, may put a cell at a growth disadvantage whereas loss of QS, a gene foreign to *Drosophila*, may be a neutral event. For these reasons, QS LOH cells may be more robust than *mwh* LOH cells and are, therefore, retained more efficiently into adulthood. Also, p53 appears to play a role in distinguishing the two types of cells; p53^RNAi^ reduced the retention of *mwh* LOH cells by 4-fold and QS LOH cells by 1.2-2-fold. Understanding how cells with different degrees of LOH are distinguished could have important implications in understanding genome stability and diseases that result from the lack of it such as cancer. We speculate that the persistence of some LOH cells may lead to their proliferation and, in some cases, oncogenic transformation.

In conclusion, we have developed a QF/QS-based system to monitor LOH in larval, pupal and adult stages, and have used it to identify two different culling steps for cells with genome instability after irradiation. The utility of this system, in conjunction with GAL4/UAS, allowed us to identify different genetic requirements as shown in Fig 11B. We document better retention of QS LOH cells into the adult structure compared to *mwh* LOH cells; therefore, the QF/QS system may be a more sensitive assay for LOH and a good community resource.

## Materials and methods

### *Drosophila* stocks and methods

*Drosophila* stocks used in this work are described in Flybase and listed in S2 Table and include: *w*^*1118*^, psc-QF>mtdTomato; tub-QS (on Ch II & III, Bloomington stock# or BL30042), psc-QF>mtdTomato/CyO (on Ch II, BL30043), Df(3L)H99 (on Ch III, BL1576), Df(3L)X14 (on Ch III, [7, 36]), *hid*^*05014*^ (on Ch III, BL83349), UAS-Dcr (on X, from BL24644 & BL24645), *mwh*^*1*^ (on ch III, BL549), UAS-eGFP (on II, BL5431), and UAS-p53^RNAi^ (VDRC38235). QS at 100E1 is inserted within nucleotides 31985262–31985725 (Christopher Potter and Linqun Luo, personal communication), which maps to the 5’ non-coding region of *heph*. psc-QF>QUAS-Tom; tub-QS homozygotes were crossed to *w*^*1118*^ (control) or mutants with the TM6-Tb balancer. Experimental larvae were identified by the lack of Tb marker in the latter group. In experiments with Dcr; p53 RNAi, UAS-Dcr/UAS-Dcr; en-GAL4>UAS-GFP, UAS-p53RNAi/CyO virgin females were used and GFP-positive larvae or pupae or Cy^+^ adults were selected for dissection. In experiments with UAS-Dronc^DN^, psc-QF, QUAS-Tom, en-GAL4, UAS-GFP/CyO-GFP; tub-QS, tub-GAL80^ts^/TM6-Tb animals were crossed to UAS-Dronc^DN^ /TM6B-Tb animals, and larvae lacking GFP and Tb markers were used. Recombinant chromosomes were generated by standard *Drosophila* techniques: chromosome II bearing en-GAL4, UAS-GFP and UAS-p53^RNAi^; chromosome II bearing psc-QF, QUAS-Tom, en-GAL4, UAS-GFP; and chromosome III bearing tub-QS, tub-GAL80^ts^. For the induction of neutral clones, Act-FRT-GAL4>UAS-RFP (on II, BL30558) was crossed to hs-FLP (on III, BL55815). RFP+ larvae and pupae or Sb+ adults were identified and processed.

### Larvae culture and irradiation

Larvae were raised on Nutri-Fly Bloomington Formula food (Genesee Scientific) at 25°C unless otherwise noted. The cultures were monitored daily for signs of crowding, typically seen as ‘dimples’ in the food surface as larvae try to increase the surface area for access to air. Cultures were split at the first sign of crowding. Larvae in food were placed in petri dishes and irradiated in a Faxitron Cabinet X-ray System Model RX-650 (Lincolnshire, IL) at 115 kVp and 5.33 rad/sec. For eclosion, empty and full pupal cases were counted 12–14 days after irradiation.

### Heat-shock induction of non-LOH clones

Embryos were collected for 24h and aged at 25°C for 72h to produce 3-4d old larvae. Larvae were heat-shocked by placing the vial in a 37°C bath for 30 minutes, before being returned to 25°C for the remainder of the experiment.

### Tissue preparation

Larval wing discs were dissected in PBS, fixed in 4% para-formaldehyde (PFA) in PBS for 30 min, and washed three times with PBTx (0.1% Triton X-100). The discs were stained with 10 μg/ml Hoechst33342 in PBTx for 2 min, washed 3 times, and mounted on glass slides in Fluoromount G (SouthernBiotech). For Acridine Orange staining, Larvae were dissected in PBS, and imaginal discs were incubated for 5 min in PBS plus 0.5 mM AO (Sigma) at room temperature, washed twice with PBS, mounted in PBS, and imaged immediately.

To obtain pupal wings, white pupae were collected as they formed on the side of the culture bottles/vials (0h APF) and were placed on a strip of double-sided tape in a petri dish with dorsal sides up and cephalic ends pointing in the same direction. The pupae were aged for 24-36h before the operculum was removed with forceps. The resulting opening was used to start an incision along the lateral side of the pupal casing extending from the head to the caudal end. The incision was made with forceps while illuminating the specimen to see the internal shape of the pupa, with care taken to not puncture the wing epithelium. The pupal casing was peeled over and stuck to the tape starting at the anterior end of the lateral incision leaving a nearly “naked” pupa. The “naked” pupa was gently transferred to a second piece of double-sided tape and fixed in 100 μl of 8% PFA in PBS for 5 min. The wing cuticle sac was removed by peeling from the hinge region to the wing tip. The wing was excised and transferred to a round bottom well in a 9-well glass plate containing 100 μl of 8% PFA in PBS to fix for 5 min, rinsed with 100 μl PBTx, stained in 100 μl of 10 μg/ml Hoechst33342 in PBTx for 3 min, and rinsed in 100 μl of PBTx. The wings were mounted on a microscope slide in 20 μl of Fluoromount-G (SouthernBiotech).

For adult wings in QF/QS experiments, newly eclosed adults that have yet to unfurl wings were collected and kept at room temperature until the wings unfurl. The wings were collected within 30 min of unfurling, submerged in PBTx, mounted in Fluoromount G (SouthernBiotech), and imaged immediately. Any delay, we found, meant the loss of fluorescent signal as wing epithelial cells are eliminated in a developmentally regulated wing maturation step [37, 38]. This protocol allowed us to image wings before the elimination step as confirmed by the GFP signal in the posterior half of adult wings in en-GAL4>UAS-GFP, UAS-p53^RNAi^ experiments. Adult wings in *mwh* experiments were collected without regard to timing. Male and female adults were recovered in as expected in Mendelian ratios. Therefore, each dataset, on average, is comprised of an equal number of male and female wings.

### Image analysis

*Drosophila* tissues were imaged on a Leica DMR compound microscope using a Q-Imaging R6 CCD camera and Ocular or Micro-Manager software. For analysis of pupa and adult wings, we express LOH area as % of total wing area. Normalizing to total wing area was done to correct for variations in wing size from individual to individual, any differences in the degree of tissue flattening while mounting pupal wings, and difference in wing size between adult males and females. In support, re-analysis of adult wing data for a single sex reproduced the difference between control and *Srp*^*neo45*^*/+* wings analyzed without separating the sexes (S1 Fig). In QF/QS experiments, Tom signal was obtained from both sides of pupa and adult wings using different focal planes. As such % area was computed by dividing with 2X the wing surface area from one side. In *mwh* experiments, LOH cells were counted from only the upper wing layer. Therefore, % area or LOH cells/area were computed using 1X wing surface area. In UAS-RFP experiments (Fig 6), RFP+ area was computed from one layer of the pupa and adult wings using 1X wing surface area.

### Statistical analysis

For sample size justifications, we used a simplified resource equation from [39]; E = Total number of animals − Total number of groups, where E value of 10–20 is considered adequate. When we compare two groups (*QF>Tom/+;QS/+ vs QF>Tom/+;QS/H99*, for example, where the former is the control group and the latter is the treated group), n = 6 per group or E = 11 would be adequate. All samples subjected to statistical analysis meet or exceed this criterion. For comparison of binned populations (Figs 2–3, 8), Kolmogorov–Smirnov test was used to calculate p-values. For other comparisons, 2-tailed Student t-tests were used.

## Supporting information

S1 Fig Tom area in adult male flies The dataset in Fig 8F was re-plotted for just males. The greater Tom area seen in *Srp/+* wings in Fig 8F is also seen here. Low number of females in the *QF>Tom/+; QS/+* dataset prevented a similar analysis.
(TIF)

S1 Table p-values. (DOCX)

S2 Table Fly stocks. (DOCX)
