# Supplementary figures and images for: Cells with loss-of-heterozygosity after exposure to ionizing radiation in Drosophila are culled by p53-dependent and p53-independent mechanisms
Source: PLoS Genet. 2020 Oct 19;16(10):e1009056. doi: 10.1371/journal.pgen.1009056 (PMC7595702; doi:10.1371/journal.pgen.1009056)

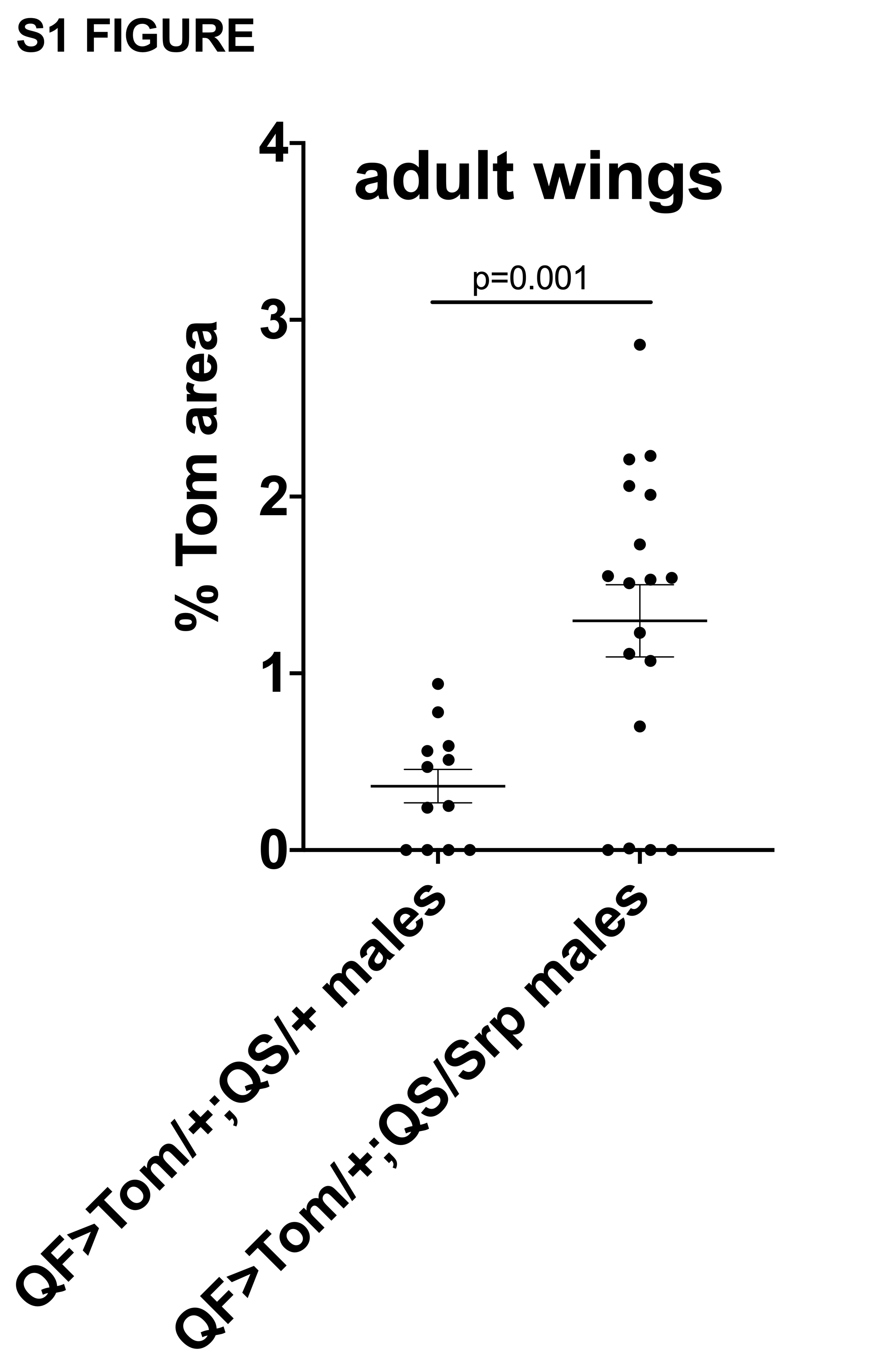

Supplement: S1 Fig — The greater Tom area seen in Srp/+ wings in Fig 8F is also seen here. Low number of females in the QF>Tom/+; QS/+ dataset prevented a similar analysis. (TIF) [file pgen.1009056.s001.tif]
