# Supplementary material for: Cells with loss-of-heterozygosity after exposure to ionizing radiation in Drosophila are culled by p53-dependent and p53-independent mechanisms
Source: PLoS Genet. 2020 Oct 19;16(10):e1009056. doi: 10.1371/journal.pgen.1009056 (PMC7595702; doi:10.1371/journal.pgen.1009056)
Supplement: S1 Table — (DOCX) [file pgen.1009056.s002.docx]

| **p-values from Kolmogorov–Smirnov test, for binned samples** | | | | | | |
| --- | --- | --- | --- | --- | --- | --- |
| **Fig.** | **Experiment** | **Sample 1** | **n=** | **Sample 2** | **n=** | **p-value** |
| 2L | IR+48h | QF;QS 0R | 38 | QF;QS 1000R | 23 | 3.41E-05 |
| 2L | IR+48h | QF;QS 1000R | 19 | QF;QS 4000R | 32 | 2.33E-03 |
| 2L | 4000R+48h | QF;QS 3-4d old | 32 | QF;QS 4-5d old | 45 | 2.43E-04 |
| 2L | 4000R+72h | QF;QS 3-4d old | 40 | QF;QS 4-5d old | 24 | 4.58E-02 |
| 2L | QF;QS 3-4d old | 4000R+48h | 32 | 4000R+72h | 40 | 2.36E-04 |
| 2L | QF;QS 4-5d old | 4000R+48h | 45 | 4000R+72h | 24 | 1.42E-02 |
|  |  |  |  |  |  |  |
| 3G | 3-4d old 4000R+48h | QF;QS | 32 | H99/+ | 52 | 1.68E-08 |
| 3G | 3-4d old 4000R+72h | QF;QS | 40 | H99/+ | 83 | 1.08E-06 |
| 3G | 3-4d old 4000R+72h | QF;QS | 40 | hid^05014^/+ | 56 | 2.33E-02 |
| 3G | 3-4d old 4000R+72h | QF;QS | 40 | X14/+ | 44 | 0.348 |
| 3G | H99/+ 3-4d old 4000R | IR+48h | 52 | IR+72h | 83 | 1.08E-07 |
| 3G | hid^05014^/+ 3-4d old 4000R | IR+48h | 57 | IR+72h | 56 | 0.104 |
| 3G | X14/+ 3-4d old 4000R | IR+48h | 32 | IR+72h | 44 | 0.968 |
|  |  |  |  |  |  |  |
| **p-values from 2-tailed Student t-test, for non-binned samples** | | | | | | |
| 2M | IR+48h | QF;QS 0R | 38 | QF;QS 1000R | 17 | 2.41E-06 |
| 2M | IR+48h | QF;QS 1000R | 17 | QF;QS 4000R | 32 | 4.29E-03 |
| 2M | QF;QS 3-4d old | 4000R+48h | 32 | 4000R+72h | 39 | 0.831 |
| 2M | QF;QS 4-5d old | 4000R+48h | 32 | 4000R+72h | 23 | 0.108 |
|  |  |  |  |  |  |  |
| 3H | 3-4d old 4000R+72h; larva area | QF;QS | 39 | H99/+ | 65 | 3.18E-03 |
| 3H | 3-4d old 4000R+72h; larva area | QF;QS | 39 | hid^05014^/+ | 50 | 0.637 |
| 3H | 3-4d old 4000R+72h; larva area | QF;QS | 39 | X14/+ | 41 | 0.047 |
|  |  |  |  |  |  |  |
| 10M | p53^RNAi^ clone number; larva IR 48h | anterior | 38 | posterior | 38 | 0.753 |
| 10M | p53^RNAi^ clone number; larva IR 72h | anterior | 19 | posterior | 19 | 0.132 |
| 10N | p53^RNAi^ clone number; anterior | pupa | 16 | adult | 19 | 0.876 |
| 10N | p53^RNAi^ clone number; posterior | pupa | 16 | adult | 19 | 0.074 |
| 10O | p53^RNAi^ clone area; anterior | pupa | 16 | adult | 19 | 0.049 |
| 10O | p53^RNAi^ clone area; posterior | pupa | 16 | adult | 19 | 0.146 |

**S1 Table. p-values. ‘**QF;QS’ = QF>Tom/+; QS/+
