# Supplementary material for: Cells with loss-of-heterozygosity after exposure to ionizing radiation in Drosophila are culled by p53-dependent and p53-independent mechanisms
Source: PLoS Genet. 2020 Oct 19;16(10):e1009056. doi: 10.1371/journal.pgen.1009056 (PMC7595702; doi:10.1371/journal.pgen.1009056)
Supplement: S2 Table — (DOCX) [file pgen.1009056.s003.docx]

| **Source (BL = Bloomington Stock Center)** | **Used in Fig.** | **Genotype** |
| --- | --- | --- |
| BL30042 | 1-5, 7-8, 10 | y[1] w[1118]; P{w[+mC]=M2ET-QF}ET40, P{w[+mC]=QUAS-mtdTomato-3xHA}CP; P{ry[+t7.2]=neoFRT}82B P{w[+mC]=tub-QS.P}9B |
| BL30043 | 1 | y[1] w[1118]; P{w[+mC]=QUAS-mtdTomato-3xHA}14 P{w[+mC]=M2ET-QF}ET40/CyO or ‘QF>Tom’ |
| BL1576 | 3, 7 | Df(3L)H99/TM6B-TB |
| BL83349 | 3, 7 | P{ry[+t7.2]=PZ}hid[05014]/TM6B, Tb |
| Ref. 7, 8 | 3 | Df(3L)X14/TM6B-TB |
| Ref. 23 | 4 | UAS-Dronc^DN^/TM6B-Tb, on chromosome III |
| BL 30564 | 4, 9, 10 | *engrailed-*GAL4 on chromosome II, P{en2.4-GAL4}e16E |
| Bloomington | 4, 9, 10 | *tub-GAL80^ts^* on chromosome III |
| BL5431 | 4, 9,, 10 | UAS-eGFP on chromosome II |
| BL30558 | 6 | w[1118]; P{w[+mC]=GAL4-Act5C(FRT.CD2).P}S, P{w[+mC]=UAS-RFP.W}3/TM3, Sb[1] |
| BL55815 | 6 | w[1118] P{y[+t7.7] w[+mC]=hs-FLPD5}attP3 |
| BL7609 | 7 | w[1118]; Df(3L)Exel6130, P{w[+mC]=XP-U}Exel6130/TM6B, Tb[1] |
| BL59020 | 8 | w[*]; P{hsneo}srp[neo45] e[1]/TM6B, Tb[1] |
| BL549 | 9 | mwh[1] |
| BL24644 | 9, 10 | P{w[+mC]=UAS-Dcr-2.D}1, w[1118]; Pin[1]/CyO |
| BL24645 | 9, 10 | P{w[+mC]=UAS-Dcr-2.D}1, w[1118]; Df(3L)Ly, sens[Ly-1]/TM3, Sb[1] |
| VDRC38235 | 9, 10 | w1118; P{GD4483}v38235 (dsRNA against dp53) |

**S3 Table. Fly stocks used**
